# Significance of Heme and Heme Degradation in the Pathogenesis of Acute Lung and Inflammatory Disorders

**DOI:** 10.3390/ijms22115509

**Published:** 2021-05-24

**Authors:** Stefan W. Ryter

**Affiliations:** Proterris, Inc., Boston, MA 02118, USA; Stefan.Ryter@proterris.com

**Keywords:** acute lung injury, carbon monoxide, heme, heme oxygenase, inflammation, lung disease, sepsis

## Abstract

The heme molecule serves as an essential prosthetic group for oxygen transport and storage proteins, as well for cellular metabolic enzyme activities, including those involved in mitochondrial respiration, xenobiotic metabolism, and antioxidant responses. Dysfunction in both heme synthesis and degradation pathways can promote human disease. Heme is a pro-oxidant via iron catalysis that can induce cytotoxicity and injury to the vascular endothelium. Additionally, heme can modulate inflammatory and immune system functions. Thus, the synthesis, utilization and turnover of heme are by necessity tightly regulated. The microsomal heme oxygenase (HO) system degrades heme to carbon monoxide (CO), iron, and biliverdin-IXα, that latter which is converted to bilirubin-IXα by biliverdin reductase. Heme degradation by heme oxygenase-1 (HO-1) is linked to cytoprotection via heme removal, as well as by activity-dependent end-product generation (i.e., bile pigments and CO), and other potential mechanisms. Therapeutic strategies targeting the heme/HO-1 pathway, including therapeutic modulation of heme levels, elevation (or inhibition) of HO-1 protein and activity, and application of CO donor compounds or gas show potential in inflammatory conditions including sepsis and pulmonary diseases.

## 1. Introduction

Heme (iron protoporphyrin-IX) is a naturally occurring iron chelate that exerts vital functions in cellular and organismic homeostasis, and which paradoxically can also play deleterious roles in organ pathophysiology [1,2,3,4]. Thus, the biological synthesis, utilization, and turnover of heme are tightly regulated [5,6]. The synthesis of heme begins and ends in the mitochondria, where heme-containing cytochromes exert indispensable functions in cellular bioenergetics as components of the electron transport chain (ETC) [7,8]. Additionally, heme serves as a prosthetic group in proteins involved in oxygen transport and storage, cellular and xenobiotic metabolism, cell signaling, and transcriptional regulation [8,9]. Genetic deficiencies in heme synthesis pathway enzymes are associated with inherited human diseases (e.g., porphyrias) [10,11]. In addition to physiological functions, heme in excess or released in certain pathophysiological contexts can have a pro-injury role in inflammatory diseases [1]. These effects of heme are primarily attributed to potential pro-oxidant effects, involving iron-dependent catalysis of free radical generating reactions [12]. In addition to oxidative stress, heme can modulate inflammation and innate immune programs [1]. Further, heme and heme-iron dependent cytotoxicity, may be associated with the activation of programmed cell death pathways, including apoptosis, necroptosis, and ferroptosis [13,14,15,16,17,18]. Importantly, circulating heme released from hemoglobin during hemolytic disorders, can injure vascular endothelium, leading to compromised vascular function [19,20,21,22]. In human diseases, free heme has been implicated as a harmful mediator in sickle cell disease (SCD) [23,24,25,26,27], malaria [14,28,29], sepsis [30], acute lung injury (ALI) [31,32] acute kidney injury (AKI) [33,34,35], and other inflammatory conditions. 

Heme is degraded by the heme oxygenase (HO; EC 1:14:14:18) enzyme system [36,37]. The HO system was initially characterized in 1968 as a coupled NADPH-dependent microsomal oxygenase system distinct from cytochrome p-450-dependent drug metabolism but nevertheless requiring the reductase component of cytochrome p-450 [36,37]. In the HO reaction, heme serves as its own catalytic co-factor in its enzyme-dependent oxygenation [38]. Molecular genetics characterizations have revealed two distinct molecular species: heme oxygenase-1 (HO-1) and heme oxygenase-2 (HO-2), each encoded by distinct genes [39]. HO-2 is regarded as primarily constitutively expressed, whereas HO-1 represents the inducible form [4].

HO-1, in addition to providing essential metabolic function, represents a major inducible component of cellular defense as a principal player in the mammalian stress response [40]. HO-1 was identified as a stress protein whose regulation responded to chemical and physical perturbations including its enzymatic substrate heme, and other pro-oxidant compounds (e.g., H_2_O_2_, menadione), heavy metals and thiol-reactive substances, polyphenolic antioxidants, heat stress, ultraviolet-A radiation and altered oxygen (O_2_) environments [41,42,43,44]. Although genetic deficiency of HO-1 is extremely rare in humans, clues to the systemic importance of HO-1 were revealed in the initial characterization of a single documented case. The individual with HO-1 deficiency presented with severe growth retardation, abnormal coagulation/fibrinolysis and evidence of extensive endothelial cell damage. Interestingly, the subject also presented with anemia, and pathological redistribution of iron in tissues, including the kidneys [45]. The importance of HO-1 in systemic homeostasis and iron metabolism has also been validated in studies using mice genetically-deficient in HO-1 (*Hmox1^−/−^*). These mice display a phenotype of increased oxidative stress, and abnormal systemic iron metabolism as evidenced by hepatic and renal iron deposition (localized to renal cortical tubules, Kupffer cells, hepatocytes, and hepatic vascular tissue) and serum iron-deficiency anemia [46]. Furthermore, embryonic fibroblasts isolated from these mice were sensitized to oxidant and heavy metal-induced toxicity [47]. An important role for HO-1, and of HO-1-dependent heme degradation, in the regulation of inflammation, emerged from studies that discovered a protective function of HO-1 in limiting macrophage inflammatory responses. Specifically, HO-1 was associated with attenuation of Toll-like receptor-4 (TLR4)-dependent pro-inflammatory cytokines production in lipopolysaccharide (LPS)-stimulated macrophages [48]. HO-1 expression was also associated with cytoprotection, including attenuation of TNF-α-mediated apoptosis in fibroblasts and endothelial cells [49,50].

Heme-derived CO, generated endogenously by HO activity, or applied at low concentrations designed to mimic biological production, may impact cellular functions by affecting endogenous signal transduction pathways [51,52]. Specifically, these effects include the modulation of apoptosis and other regulated forms of cell death, inflammation, cell proliferation, autophagy and other biological processes [51,52,53]. Collectively, these studies establishing cellular effects of CO served as the basis for widespread development of CO releasing molecules (CORMs) and organic CO-donor compounds, as potential candidates for therapeutic application [54,55]. Further, these studies set the stage for current and projected clinical studies to test inhaled CO (iCO) for therapeutic benefit in human subjects [52,56]. This review will explore the pro-and antioxidant sequelae of heme accumulation, and activation of its degradation pathway via HO-1 modulation, and their combined relevance to the pathogenesis of inflammatory disorders. Emphasis will be placed on ALI, sepsis, and other inflammatory conditions, with consideration of therapeutic implications.

## 2. Physiological Roles of the Heme Molecule

### 2.1. Heme Synthesis

**Figure 1:**
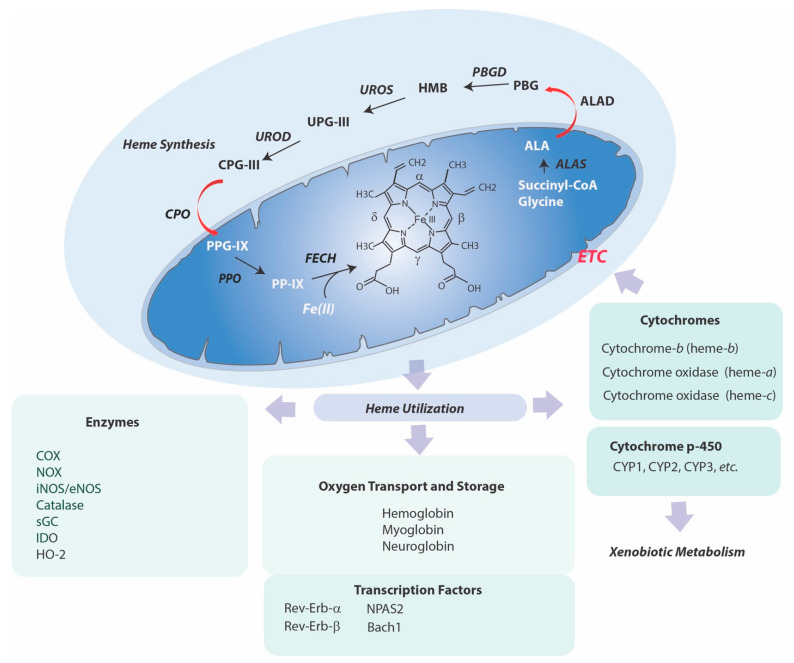
Mammalian Heme Synthesis and Utilization Pathways. Heme synthesis requires eight sequential enzymatic steps, which begin and end in the mitochondria, with intermittent cytosolic steps. (Step 1), mitochondrial 5-aminolevulinic acid synthase (ALAS; EC 2.3.1.37) condenses succinyl-Co-A and glycine to form 5-aminolevulinic acid (ALA). (Step 2) Two mol ALA are condensed to porphobilinogen (PBG) by ALA dehydratase (ALAD, EC 4.2.1.24, porphobilinogen synthase). (Step 3) Porphobilinogen deaminase (PBGD, EC 2.5.1.61, hydroxymethylbilane synthase) condenses four mol PBG, to generate hydroxymethylbilane (HMB). (Step 4) Uroporphyrinogen-III synthase (UROS, EC 4.2.1.75, uroporphyrinogen III cosynthase) catalyzes the cyclization of hydroxymethylbilane and inversion the D ring to form uroporphyrinogen III (UPGIII). (Step 5) Uroporphyrinogen III is decarboxylated by uroporphyrinogen decarboxylase (UROD, EC 4.1.1.37) to generate coproporphyrinogen III (CPGIII). (Step 6) Coproporphyrinogen III is imported into mitochondria and then decarboxylated by coproporphyrinogen oxidase (CPO, EC 1.3.3.3) to form protoporphyrinogen IX (PPGIX). (Step 7) Protoporphyrinogen IX is converted to protoporphyrin IX (PPIX) by protoporphyrinogen oxidase (PPO, EC 1.3.3.4, protoporphyrinogenase). In the final step (Step 8), ferrous iron is incorporated into the PPIX ring within the mitochondria to form heme-*b* by the enzyme ferrochelatase (FECH, EC 4.99.1.1, protoheme ferrolyase). Heme is used systemically for oxygen transport and storage functions. In eukaryotic cells, heme is utilized for peroxidase, monooxygenase, and dioxygenase activities, and for cytochromes, including cytochrome p-450s involved in drug metabolism and mitochondrial respiratory chain components.

There exists an intimate relationship between heme and the mitochondria, wherein the heme molecule originates in this organelle and also acts as an essential co-factor in bioenergetic reactions [7,8]. Heme synthesis requires eight sequential enzymatic steps, which begin and culminate in the mitochondria (step 1 and steps 7–8), with the intermediate steps (steps 2–6) occurring in the cytoplasm (Figure 1) [57]. 

In the rate limiting step of heme biosynthesis, (step 1) mitochondrial 5-aminolevulinic acid synthase (ALAS), which utilizes pyridoxal 5’-phosphate as a co-factor, condenses succinyl-Co-A derived from the tricarboxylic acid (TCA) cycle and the amino acid glycine, to form 5-aminolevulinic acid (ALA). ALAS localizes to the matrix side of the mitochondrial inner membrane. Following its mitochondrial export, ALA is condensed to porphobilinogen (step 2) by cytosolic ALA dehydratase (ALAD, porphobilinogen synthase). Porphobilinogen deaminase (PBGD) condenses four mol porphobilinogen, to generate the linear tetrapyrrole precursor hydroxymethylbilane (step 3). Uroporphyrinogen-III synthase (UROS) catalyzes the cyclization of hydroxymethylbilane with inversion the D-ring to form uroporphyrinogen III, a common precursor of tetrapyrroles (step 4). A shunt pathway generates uroporphyrinogen I, and subsequently coproporphyrinogen I, which are not utilized in heme biosynthesis. Uroporphyrinogen III is decarboxylated by uroporphyrinogen decarboxylase (UROD) to produce coproporphyrinogen III (step 5). Coproporphyrinogen III is imported into mitochondria by unknown transport mechanism, and the subsequent enzymatic steps take place at the cytosolic side of the inner mitochondrial membrane. Coproporphyrinogen III is decarboxylated by coproporphyrinogen oxidase (CPO) (step 6). The resulting protoporphyrinogen IX is then converted to protoporphyrin IX (PP-IX) (step 7) by protoporphyrinogen oxidase (PPO). In the final enzymatic step (step 8), ferrous iron is incorporated into the PP-IX ring to form heme-*b* by the enzyme ferrochelatase (FECH) on the matrix side of the inner mitochondrial membrane [58,59]. FECH consists of a homodimer, with each subunit containing and [2Fe–2S] cluster [59]. Accumulation of heme has a feedback inhibitory role on its synthesis by inhibiting ALAS1, but not the erythroid-specific form of ALAS (ALAS2) [8]. Additional enzymatic transformation of heme generates *a* and *c* type hemes used in electron transport. Heme *c* is formed by covalent attachment of heme-*b* to the cytochrome *c* apoprotein by cytochrome c-heme lyase [59]. Heme-*a* is synthesized from heme-*b* via the sequential action of heme-*o* synthase and heme-*a* synthase [60].

### 2.2. Disorders of Heme Synthesis: Porphyrias

Genetic deficiencies or mutations in heme synthesis pathway enzymes are associated with human diseases, commonly known as porphyrias [10,11]. These conditions are associated with pathological accumulations of heme precursor molecules and are broadly classed into two groups: acute hepatic porphyrias (AHPs) [10] and cutaneous porphyrias [11]. Among the AHPs, deficiency in ALAD (step 2) results in ALAD-deficiency porphyria, a rare autosomal-recessive disorder. PBGD deficiency (step 3) results in acute intermittent porphyria, associated with urinary accumulations of ALA and porphobilinogen. CPO deficiency (step 6) results in hereditary coproporphyria; and deficiency in PPO (step 7) results in variegate porphyria. ALA, which accumulates in these disorders, is believed to represent the primary neurotoxic agent [10]. Among the cutaneous porphyrias, which are associated with skin ailments and photosensitivity, UROS deficiency (step 4) results in congenital erythropoietic porphyria, with increased production of uroporphyrinogen I and toxic accumulation of coproporphyrinogen I. UROD deficiency (step 5) results in porphyria cutanea tarda, with cutaneous photosensitivity associated with uroporphyrinogen III buildup. Genetic deficiency of FECH (step 8) results in erythropoietic porphyria. Accumulation of PP-IX in this disorder results in severe skin photosensitivity [11], ALAS2 (step 1) gain-of-function mutations result in X-linked porphyria, which, similar to erythropoietic porphyria, is also associated with cutaneous PP-IX accumulation and skin photosensitivity [11,61]. Mutations in ALAS2 are also associated with X-linked sideroblastic anemia [61,62].

## 3. Heme Degradation

### 3.1. Heme Oxygenases

**Figure 2:**
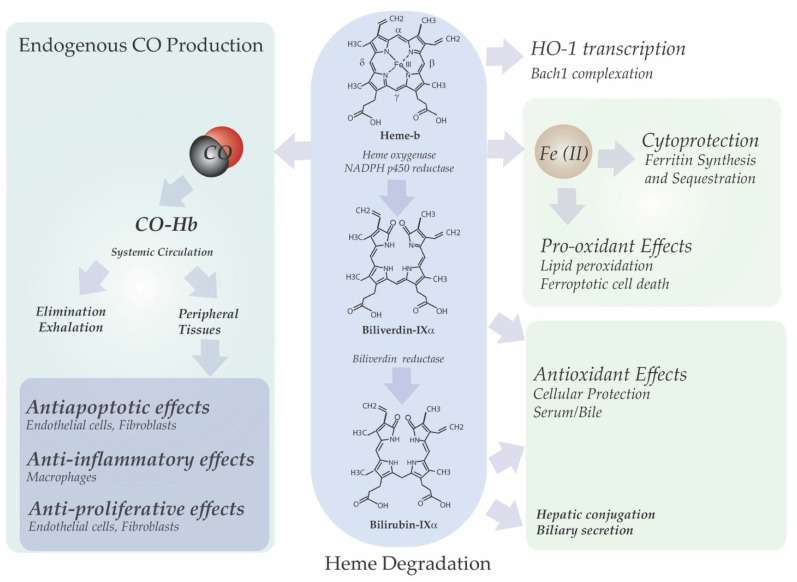
Heme degradation and cytoprotective effects of the reaction products. The heme molecule is a potent transcriptional activator of heme oxygenase-1 via repression of the transcriptional inhibitor Bach1, resulting in increased synthesis of enzymatically active. The heme oxygenase (HO, EC 1:14:14:18) reaction oxidizes heme, which serves as substrate and co-factor in its degradation, at the α-methene bridge carbon. The reaction, which requires O_2_, NADPH and the reductase component of cytochrome p450, produces carbon monoxide (CO), biliverdin-IXα and ferrous iron (Fe II). In the second step of heme degradation biliverdin-IXα is reduced to bilirubin-IXα by NAD(P)H: biliverdin reductase (BVR; EC 1.3.1.24). Both BV and BR are implicated as cellular and circulating antioxidants. Iron released from HO activity is sequestered in a complex with ferritin, which serves as a cellular antioxidant. Excess iron may drive pathological processes including free radical generation and ferroptotic cell death. CO generated from the HO reaction can exert multiple cellular effects, which may be beneficial at low concentrations. Namely, these include inhibition of apoptosis and inflammatory pathways, as well as inhibition of cell proliferation. CO forms a tight binding complex with hemoglobin in circulation to form carboxyhemoglobin (CO-Hb). CO is eliminated by diffusion at the alveolus and exhaled.

Heme turnover in mammals is catalyzed by microsomal heme oxygenase (HO) [EC 1:14:14:18, decyclizing] [36,37]. HO catalyzes the oxygenation of heme at the α-methene bridge carbon, to generate carbon monoxide (CO), and biliverdin-IXα (BV) as the products of tetrapyrrole cleavage, while releasing the central heme iron chelate as ferrous iron (Fe-II) (Figure 2). Enzymatic heme degradation utilizes molecular oxygen (O_2_) and electrons derived from NADPH: cytochrome p450 reductase (EC 1.6.2.4) [36,37]. The three oxygenation cycles proceed via three intermediates: a *meso*-hydroxyhemin, verdoheme, and a ferric iron-biliverdin complex [38]. The BV generated in the HO reaction is reduced to bilirubin-IXα (BR) by cytosolic NAD(P)H: biliverdin reductase (BVR; EC 1.3.1.24) [63]. BR, which is lipid soluble is conjugated in the liver by uridine diphosphate glycosyltransferase 1A1 (UGT1A1; EC 2.4.1.17) and eliminated via the biliary fecal route [64,65]. HO consists of two major isoforms, an inducible isozyme (HO-1) and a constitutively expressed isozyme (HO-2), which are encoded by distinct genes [4,39]. Heme is the natural substrate of HO-1 and HO-2 [4]. 

Although HO-1 is not a hemoprotein *per se*, it becomes a transient hemoprotein upon binding to the catalytic site, whereby heme catalyzes its own oxygenation [66]. HO-2 binds heme at its catalytic site but also bears two additional heme binding sites termed heme regulatory motifs (HRMs), which contain Cys-Pro motifs [67]. In recent studies, mutation in the HRMs of HO-2 was found to accelerate HO-2 turnover via chaperone-mediated autophagy, suggesting that heme bioavailability regulates post-translational stability of HO-2 [68]. Ferric heme bound to HRMs may be reversibly transferred to the HO-1 catalytic site for degradation [69]. Redox-dependent variation in the modes of heme ligand orientation to HRMs of HO-2 and other hemoproteins, suggest that these domains participate in redox sensing mechanisms [70].

### 3.2. Disorders of Heme Degradation (Neonatal Jaundice, Hyperbilirubinemia)

Excess hepatic HO activity in neonates leads to serum hyperbilirubinemia [71]. Mild elevations in unconjugated serum bilirubin are associated with reduction of cardiovascular disease (CVD) risk, where high serum bilirubin may pose a risk for neurological sequelae, leading to acute and chronic (kernicterus) bilirubin encephalopathy [71,72]. Phototherapy to remove bilirubin remains the current clinical mainstay [73,74]. Treatment with metalloporphyrin inhibitors (i.e., SnPPIX) have been explored as alternate therapy [75]. In contrast, genetic deficiency of HO-1 is an extremely rare condition associated with endothelial cell dysfunction and severe cardiovascular abnormalities [45].

## 4. Heme Utilization

### 4.1. Heme Utilization in Oxygen Transport

Heme serves as a prosthetic group in vital cellular hemoproteins involved in oxygen transport and storage [8]. Heme is the cofactor for hemoglobin, the principal oxygen carrier in the blood. The iron center of heme-*b* is coordinated to four nitrogens of the porphyrin ring and one amino acid of the apoprotein, leaving one coordination site available for gaseous ligand binding [76]. Heme also serves as prosthetic group for myoglobin, the oxygen storage component of muscle [77]. Neuroglobin, which contains a single globin monomer with hexacoordinated heme, serves as an oxygen carrier in neurons with the ability to prevent hypoxic injury [78].

### 4.2. Heme Utilization in Cellular Processes

Heme-*b* and its derivatives (heme-*a* and heme-*c*) serve as prosthetic co-factor in mitochondrial respiratory chain complexes (complex II, complex III, complex IV, and cytochrome *c*) [79]. Heme is used as cofactor for essential enzymatic activities, including peroxidases, monooxygenases, and dioxygenases. For example, heme serves as cofactor for cytochrome p-450s, which are a family of monooxygenases involved in drug metabolism and steroid biosynthesis. Heme serves as a cofactor for prostaglandin cyclooxygenase, and of indoleamine 2,3-dioxygenase 1 (IDO1), which converts tryptophan to kynurenine [80,81]. Additionally, catalase, which catalyzes the decomposition of hydrogen peroxide to water, contains four heme catalytic groups. Heme is also a co-factor of endothelial (eNOS) and inducible (iNOS) nitric oxide synthases, which catalyze the formation of nitric oxide (NO) from arginine [82]. Finally, key signaling proteins such as guanylate cyclase (sGC), which generates cGMP, has a heme moiety for sensing of gaseous ligands (i.e., NO, CO) [4].

Heme may also serve as a prosthetic or interactive group for several transcription factors [62]. The classical example, neuronal PAS domain protein 2 (NPAS2), forms heterodimers with Bmal1 involved in regulation of circadian rhythm [83]. Heme also binds to transcription factors Rev-Erb-α/β, which act as transcriptional repressors by recruiting co-repressor NCoR and histone deacetylase-3 (HDAC3) to their target gene promotors. [84,85]. Rev-Erb-α acts as a repressor of Bmal1 and PGC-1α genes, the latter which is a primary regulator of mitochondrial biogenesis [84]. Heme acts as an inhibitor of Bach1 and Bach2, of which Bach1 regulates globin gene expression and acts a repressor of the *Hmox1* gene (see section on HO-1 gene regulation below) [86]. Emerging evidence suggests that heme also regulates the processing of non-coding RNAs, including micro RNAs (miRs) involved in gene expression [62]. Additionally, other potential roles for heme in nucleic acid activities and chromatin regulation have been uncovered. For example, heme binds to guanine rich regions of DNA and RNA with high affinity, to form secondary structure termed G-quadruplexes (G4) [87]. Recent studies demonstrate peroxidase catalytic activity of heme-bound G4 DNA [88].

### 4.3. Heme Export and Scavenging

Heme is exported from cells by the feline leukemia virus subgroup C receptor-a isoform (FLVCR1a) [89]. A mitochondrial isoform FLVCR1b regulates erythropoiesis by controlling intracellular heme efflux from the mitochondria to the cytoplasm [90]. Hemopexin is the major vehicle for the scavenging and transportation of heme in the plasma for delivery to sites of detoxification (i.e., liver) [91]. Hemopexin is a plasma glycoprotein that binds heme in a 1:1 molar ratio, with the highest affinity (K_d_ < 1 pM), relative to other circulating proteins with capacity to bind heme. The function of the heme-hemopexin interaction is primarily antioxidative, in that it prevents circulating heme from catalyzing harmful reactions [91]. Other circulating proteins (i.e., albumin, α-macroglobulin) play a lesser role in heme scavenging.

## 5. Pathological Properties of Heme

### 5.1. Heme as Catalyst in Pro-Oxidant Reactions

**Figure 3:**
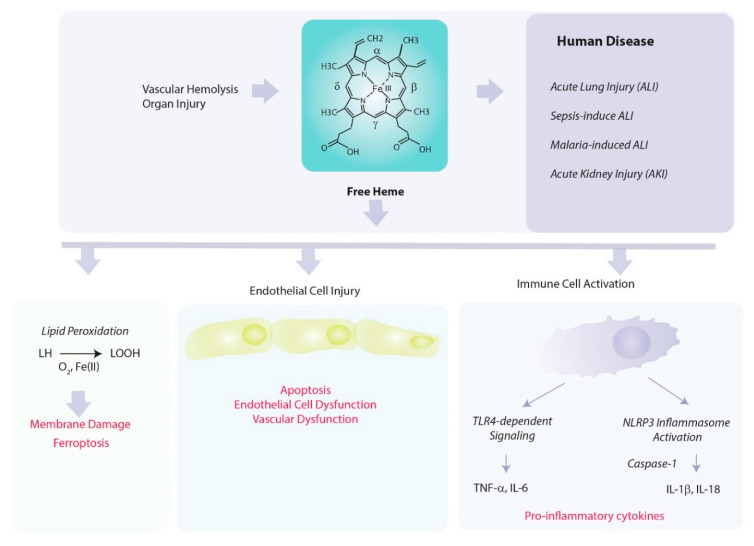
Pathological consequences of heme release. Free heme can promote hemolysis of red cells. Heme released into the circulation under hemolytic conditions poses a risk to the vascular endothelial cells, including the promotion of membrane damage, and necrotic and apoptotic cell death. Injured endothelial cells may compromise vascular function, and release DAMPs into the circulation. The toxicity of free heme may be limited by scavenging with hemopexin. Heme can act as a catalyst for pro-oxidant reactions, including LDL oxidation, and the peroxidation of lipids. Iron released from heme degradation may, in excess, further propagate pro-oxidant reactions and trigger ferroptotic cell death. Heme may also promote inflammation by activating TLR4-dependent and inflammasome-dependent pathways in inflammatory cells, leading to the production of pro-inflammatory cytokines.

Heme, while indispensable as a biological catalyst, also has a potential for acting as a harmful mediator of inflammation and disease (Figure 3). 

Heme in protein-bound form, as in hemoproteins, can form higher valence states of iron involved in oxidative catalysis [92]. In its unbound form, when released in circulation, heme can destabilize red cell integrity and thereby act a hemolytic agent [13]. The role of heme in the decomposition of peroxides has been described in the literature since the middle of the twentieth century [93,94]. Heme, as an iron chelate, can potentially catalyze iron-dependent free radical generating reactions, and has been implicated as a Fenton reagent [95]. The reaction of hydrogen peroxide with hemes, dependent on valence state, may result in formation of a porphyrin cation radical, or catalyze the decomposition of peroxide to form hydroxyl radical (**^.^**OH), a reactive species capable of oxidizing DNA, lipids, and proteins [96]. The potential for heme in accelerating lipid peroxidation has been described since the 1950s [96,97,98]. The catalysis of lipid peroxidation by heme was shown to be reversed by hemopexin binding [99]. Heme can also cause oxidation of proteins leading to cross-linking [96], and was also shown to act as a catalyst for protein nitration [100]. Further, heme transferred from methemoglobin can promote the oxidation of low-density lipoprotein (LDH), by binding to high affinity sites in the ApoB moiety, leading to generation of cytotoxic products [101,102]. By these mechanisms, heme was implicated as a pro-oxidant hazard to vascular endothelial cells, and as an initiating factor in atherogenesis [22,103].

### 5.2. Heme as a Regulator of Inflammation and Vascular Permeability

Heme has emerged as a regulator of the innate immune system. Unbound heme can activate endothelial cells and promote inflammatory responses of macrophages and neutrophils [1,104,105]. Heme causes vascular permeability in vivo and activates endothelial cells to produce intracellular adhesion molecules and secrete chemokines for neutrophil recruitment [106]. Circulating heme can be regarded as a damage associated molecular pattern (DAMP) with respect to activation of innate immune responses.

Heme acts as a regulator of Toll-like receptor-4 (TLR-4) signaling via MyD88 to regulate pro-inflammatory cytokine (i.e., TNFα) production in macrophages and other target cells [107]. A combination of antioxidant-sensitive pathways triggered by heme-dependent ROS production via spleen tyrosine kinase (*Syk*) signaling, and TLR4 activation were required for cytokines/chemokines production in macrophages and lethal effects of hemolysis in mice [108]. Recent studies describe differential effects of pro-inflammatory stimuli (i.e., LPS) on labile heme pools and *Hmox1* gene expression in mouse bone marrow-derived macrophages (BMDMs) and human monocyte-derived macrophages (MDMs). LPS induced the levels of labile heme available for upregulation of HO-1 in mouse BMDMs. In contrast, in human MDMs, LPS decreased the levels of labile heme and downregulated HO-1. The effects of LPS on the labile heme pool in macrophages were dependent on TLR4 [109]. 

Myeloid differentiation factor-2 (MD-2) is an adaptor for TLR4-dependent pro-inflammatory signaling. Recent studies have characterized a heme binding contact points (W23 and Y34) on MD2, which may facilitate heme dependent TLR4 activation [110]. Heme was also found to bind to the soluble form of MD2 (sMD2), found in plasma [111]. Heme induces IL-1β production through the activation of the nucleotide-binding domain and leucine-rich repeat-containing family and pyrin domain containing 3 (NLRP3), which activates caspase-1 in macrophages, leading to maturation and secretion of pro-inflammatory cytokines. The activation of the NLRP3 inflammasome by heme required NOX2-dependent ROS formation, and K^+^-efflux [112]. In addition to caspase-1, heme-dependent IL-1β activation was also found to require a non-canonical pathway involving caspase-4 and caspase-5 [113].

Heme may act as a potent activator of NLRP3 upregulation and IL-1β production in endothelial cells, effects which could be amplified by LPS priming [114]. Additionally, heme interaction with the receptor for advanced glycation end products (RAGE) can promote production of TNF-α and IL-1β [115].

Heme was also found to trigger endothelial barrier dysfunction by a pathway involving MKK3/p38 MAPK activation, independently of TLR4. Heme reduced the expression of tight junction proteins in human microvascular endothelial cells and upregulated HSP27 by this mechanism. These effects were reversed by genetic deficiency of MKK3 [116]. Further studies revealed that heme and serum derived from human SCD serum could induce permeability changes in human endothelial cells, and that the combined effect of SCD serum with heme was associated with low levels of hemopexin in SCD serum [117].

Recent studies also implicate heme in the regulation of platelet activation. Heme was found to bind directly to C-type lectin-like receptor-2 (CLEC-2) and glycoprotein VI (GPVI) to mediate platelet activation [118]. Heme can play a pro-inflammatory role in the context of hemolytic disorders such as SCD, malaria, and propagate neuronal injury during intracerebral hemorrhage [25,28,119]. Heme-dependent TLR4 activation was linked to pathological effects in SCD, including endothelial cell activation and vaso-occlusion [25,120]. Finally, heme induced neuroinflammation may promote the pathology of neurodegenerative disorders [121]. 

### 5.3. Heme in the Initiation of Regulated Cell Death Pathways

Data from diverse experimental model systems implicate the pathological accumulation of heme in cytotoxicity, associated with the regulation and/or activation of programmed cell death pathways. Although the regulatory mechanisms by which heme can trigger programed cell death pathways remain incompletely defined, heme has been implicated in model systems as an activator of apoptosis, necroptosis, and ferroptosis.

Apoptosis is a type of programmed cell death (PCD) that is associated with DNA fragmentation and activation of caspases. In contrast, necrosis is defined as an accidental non-regulated lytic form of cell death [122,123]. Necroptosis is defined as a genetically-regulated form of cell death, also distinct from apoptosis [124]. Similar to necrosis, necroptosis is associated with organelle swelling, plasma membrane rupture, and cell lysis. Necroptotic cells may propagate inflammatory responses via release of DAMPs. The necroptosis pathway can be activated by stimulation with death-receptor ligands. Necroptosis is regulated by receptor-interacting protein kinases-1 and -3 (RIPK1, RIPK3), and mixed lineage kinase domain-like pseudokinase (MLKL), which assemble to form a multimeric complex termed the “*necrosome*”. The phosphorylation of MLKL by RIPK3 is considered as a primary trigger for necroptosis activation [125]. In contrast to necroptosis, an iron dependent form of regulated cell death termed “*ferroptosis*”, is triggered by iron-dependent membrane lipid peroxidation [126]. 

In a mouse model of malaria, heme was implicated as a sensitizer of TNF-α-mediated apoptosis in the liver, the removal of which by hepatic HO-1 downregulated hepatic apoptosis [14]. Heme dose-dependently killed HT22 neuronal cell cultures by necroptosis, as demonstrated by inhibition with antioxidants the RIPK1 inhibitor necrostatin-1 and by RIPK3-targetted siRNA [15]. Heme was also found to trigger necroptosis in astrocytes, associated with NF-κB-dependent activation of inflammatory responses, and severe depletion of reduced glutathione (GSH) [16]. In a model of cerebral hemorrhage, heme induced ultrastructural changes in cultured neurons consistent with necrosis. Heme and hemoglobin-induced cell death could be inhibited in part with necroptosis inhibitors, and in part with ferroptosis inhibitors [17].

In macrophages, treatment with heme was observed to cause cell death associated with loss of plasma membrane integrity and exhibiting features of necrosis. Heme-induced macrophage cell death required TLR4/MyD88-dependent TNF-α production. The RIPK1 inhibitor necrostatin, or genetic deficiency in RIPK1 and RIPK3 reversed heme-induced macrophage cell death. Heme induced cell death was also reversed by JNK inhibitors and antioxidants and augmented by HO-1 deficiency [18].

HO-1, which degrades heme in exchange for free iron, has a complex relationship with ferroptosis, as both heme and iron can potentially catalyze lipid peroxidation and membrane damage. HO-1 was found to mediate erastin-induced ferroptosis in cancer cells [127]. ZnPPIX, a competitive HO-1 inhibitor, inhibited erastin-induced ferroptosis, while HO-1 induction in response to heme or CORM treatment, promoted cell death in cancer cells [127]. In a model of cancer cell ferroptosis caused by treatment with an IκBα inhibitor, cell death was associated with increased HO-1 expression, mitochondrial and nuclear translocation of HO-1 and increased mitochondrial dysfunction and elevated mitophagy [128]. In contrast to these observations, HO-1 has also been described as a protective factor against ferroptosis, following its induction by pre-conditioning agents. For example, kidney injury during rhabdomyolysis was associated with ferroptosis as shown by inhibition with ferrostatin but not RIPK3 deletion, whereas ferroptosis was reversed by induction of HO-1 with natural antioxidants [129]. HO-1 was also implicated in cellular protection against ferroptosis of kidney epithelial cells induced with erastin. Kidney proximal tubule epithelial cells derived from *Hmox1*^−/−^ mice were sensitized to erastin-mediated ferroptosis [130]. These studies, taken together, suggest that HO-1 and HO-dependent heme degradation have been implicated the regulation of ferroptosis, in a context dependent manner.

## 6. Link between HO-1 Dependent Heme Degradation and Cellular Function

### 6.1. Iron and Redox Homeostasis

The degradation of heme is intimately linked to cellular defense mechanisms, in particular via the activity of the inducible form HO-1 [12,40,41]. The primary removal of labile heme, if accumulated intracellularly, likely represents a primary antioxidative function of HO-1, in order to restrict the potential of heme for participating in harmful reactions [26]. Cellular protection conferred by HO-1 is linked to modulation of the cellular composition of redox active components (i.e., biliverdin, and iron) as well as the generation of CO (*see* section below) [12,131]. By degrading heme, HO releases heme iron, which itself can present harmful sequelae unless detoxified, including potential catalysis of Fenton chemistry, and associated production of ROS and lipid peroxides [132,133]. The release of iron by HO-1, when contributing significantly to the labile iron pool, has been proposed as a trigger for the regulation of de novo ferritin synthesis. Ferritin, a multimeric complex of H and L chains is linked to cellular defense against oxidative stress, by scavenging labile redox-active iron [134,135,136]. In this regard, ferritin has been identified as a cytoprotective molecule in the vascular endothelium [137,138,139]. Seminal studies have also linked intracellular iron accumulation to HO-deficiency, and have thus associated HO-1 activity to iron efflux mechanisms [140]. Pathological roles for HO-dependent heme iron release have also been proposed, including the context-specific promotion of ferroptotic cell death, and neuronal injury in the case of neurodegenerative disorders [141,142,143]. Additionally, HO-1 may serve as a modulator of cellular functions, via non-canonical mechanisms, independent of its enzyme activity. These include the generation of a nuclear truncated form (NHO-1), an activity-deficient isoform that can modulate transcription factor activities [144,145].

### 6.2. Bile Pigment Generation

Both BV and BR generated by HO-dependent heme degradation have been extensively characterized as antioxidants, which can attenuate free radical generating reactions in vitro, and in serum and bile. Initial characterizations implicated BR as a chain-breaking antioxidant in model systems of lipid peroxidation [146]. BR can attenuate radical damage to proteins, act as a serum antioxidant in albumin-bound form and modify redox balance in the bile [147,148,149]. Pharmacological application of BV showed benefit in murine models of organ transplant. Mild elevation of serum BR is associated with protection from CVD risk, whereas low bilirubin is associated with increased CVD risk and risk of graft rejection [71,150,151]. In addition to the enzymatic role in BV to BR conversion, biliverdin reductase has non-canonical (activity-independent) roles in cellular regulation. These attributes of BVR include Ser/Thr/Tyr kinase activity, anti-inflammatory effects via the PI3K/Akt pathway, and a nuclear form implicated in transcriptional regulation [152,153,154].

### 6.3. Heme-Derived Carbon Monoxide Production

CO released from the HO reaction has been implicated as a pleiotropic modulator of cellular functions. CO has limited biological reactivity, except for its high affinity for binding to heme iron centers. The potential targets of CO binding are, in principle, represented by cellular hemoproteins that utilize heme for catalytic activity. In this regard, CO may compete for oxygen binding to hemoglobin (with an affinity ~245 times that of oxygen), inhibit cytochrome *c* oxidase activity, and modulate the enzymatic activity of select hemoproteins, with sGC being the classic example [51,52,53]. CO was implicated as an anti-inflammatory effector molecule in macrophages based on p38 MAPK-dependent downregulation of pro-inflammatory cytokines and upregulation of the anti-inflammatory cytokine IL-10 [48]. CO was also characterized as an antiapoptotic molecule based on initial observations of inhibition of TNF-α-mediated endothelial cell apoptosis [50]. Further, evidence has accumulated that CO, when applied exogenously at low concentrations in the ppm range (i.e., 250 ppm), can confer cyto- and tissue protection in inflammatory disease models in effect by influencing inflammation, apoptosis, and cell proliferation programs [51,52,53]. The molecular and cellular effects of exogenous CO application as a therapeutic agent in animal models of injury and disease have been reviewed extensively elsewhere [51,52,53].

## 7. Regulation of HO-1 Gene Expression

The transcriptional regulation of the *Hmox1* gene encoding HO-1 is primarily operated by nuclear factor erythroid 2-related factor-2 (Nrf2) [155]. Nrf2 is a master regulator of the antioxidant response and regulates other genes involved in antioxidation and cellular detoxification, including glutathione peroxidase-2, thioredoxin, thioredoxin reductase, glutathione-S-transferases, NADPH quinone dehydrogenase 1, ferritin heavy and light chains, and other targets [156]. A member of the Cap’n’collar/basic-leucine zipper family, Nrf2 that can form heterodimers with small Maf proteins Kelch-like ECH-associated protein (Keap1) binds to Nrf2 in the cytoplasm under basal conditions [157,158]. Keap1 enables the targeting of Nrf2 by Cullin 3-based E3 ubiquitin ligase complex, which tags Nrf2 for continuous degradation by the proteasome [159,160]. In response to inducing stimuli, such as exposure of cells to polyphenolic antioxidants, Keap1 dissociates from Nrf2, which then permits Nrf2 to translocate to the nucleus, where it can transactivate *Hmox1* and other Nrf2 target genes. Heme can also inhibit the proteasomal degradation of Nrf2, thereby providing another mechanism by which heme can induce *Hmox1* gene regulation [161]. 

Transcription factor BTB and CNC homology 1 (Bach1) serves as a transcriptional repressor of HO-1 gene expression via competition with Nrf2 [162,163]. Heme forms a complex with Bach1 at its carboxy-terminal region which bears four dipeptide cysteine-proline (CP) motifs [164]. Heme binding results in inhibition of Bach1 DNA-binding activity and can also promote the nuclear export of Bach-1 thereby derepressing HO-1 expression [165,166]. Cytoplasmic Bach1 is degraded by the proteosome. Both Nrf2 and Bach1 target conserved sites located in the promoter regions of *Hmox1* genes. Nrf2 binds to consensus antioxidant response elements (ARE), and Bach1 competes with Nrf2 for occupancy at these sites. These ARE elements are found in distinct enhancer regions that occur at −4 kb and −10 kb upstream of the *Hmox1* transcriptional start site [167,168]. In addition to Nrf2, other diverse factors have been implicated in *Hmox1* regulation in a wide variety of contexts. For example, hypoxia-inducible factor-1 (HIF-1), regulates the *Hmox1* gene under conditions of cellular hypoxia [44]. Other participating transcription factors have been discussed elsewhere, and potentially include NF-κB, AP-1 and others [169,170,171]. Recent studies also implicate mIR networks in the regulation of Nrf2 and HO-1, as well as in the downstream effects of these proteins, and these mechanisms have been reviewed elsewhere [172,173]. 

## 8. Role of Heme Oxygenase in Mediating Acute Inflammation

### 8.1. Acute Lung Injury

HO-1 has emerged as a pleiotropic and multi-faceted regulator of inflammation [40,48,173,174]. Early studies have suggested a role for HO-1 in the resolution of acute inflammation. In a mouse model of acute inflammation of the pleural cavity associated with neutrophil influx, the expression of HO-1 in pleural macrophages peaked at the time of resolution. Inhibition of HO activity using Sn-protoporphyrin-IX (SnPPIX) increased inflammatory exudates in this model whereas hemin-dependent upregulation of HO-1 was observed to dampen inflammation [175].

In rodent inflammation models, LPS application induces an inflammatory response associated with ALI, pro-inflammatory cytokines production, and apoptosis of lung cells [176,177]. Intratracheal application of LPS to rats was found to elevate HO-1 expression in alveolar epithelial and lung inflammatory cells [178,179]. Preconditioning rodents with hemoglobin, as a strategy to induce HO-1, conferred lung protection and prolonged survival during LPS challenge [179]. Systemic LPS injection in rats also induced HO-1 expression in the lung and other distal organs such as intestine and heart [179]. Application of heme prior to systemic LPS challenge, which induced HO-1 in the lung, conferred anti-inflammatory protection associated with decreased circulating TNF-α levels, increased IL-10 levels, and delayed LPS-induced mortality. Conversely, inhibition of HO activity using Zn-protoporphyrin-IX (ZnPPIX) abolished the protective effect of heme-pretreatment in this model [180].

Adenoviral-directed *Hmox1* gene transfer was shown to protect against LPS-induced ALI in mice by increasing anti-inflammatory IL-10 production [181]. Overexpression of HO-1 in RAW 264.7 macrophages inhibited LPS-inducible production of pro-inflammatory cytokines in vitro [48]. *Hmox1^−/−^* mice displayed a comparable pulmonary inflammatory response to nebulized LPS challenge relative to wild-type mice but displayed increased lung dysfunction and diminished surfactant protein-B expression [182].

Akin to observations made with HO-1 modulation, application of HO-activity end products also showed therapeutic potential in LPS-induced inflammation models. Anti-inflammatory effects of low-dose inhaled CO (i.e., 250 ppm) were demonstrated in a mouse model of endotoxin shock [48]. CO preconditioning reduced the production of serum TNF-α, IL-1β whereas increased the production of IL-10; reduced organ injury and prolonged survival following LPS challenge [48]. Additionally, marked anti-inflammatory effects of CO application (250 ppm) were observed LPS-stimulated macrophages, involving downregulation of TNF-α, IL-1β, and macrophage inflammatory protein-1β (MIP-1β). These effects were attributed primarily to modulation of mitogen activated protein kinase kinase-3 (MKK3)/p38 mitogen activated protein kinase (MAPK) pathway, and other diverse signaling mechanisms [48].

The anti-inflammatory protection against LPS-induced organ injury conferred by CO was also attributed to inhibition of iNOS expression and activity in the lung, and elevation of iNOS expression and activity in the liver [183]. Pharmacological application of BV decreased pro-inflammatory cytokine production, upregulated IL-10 levels, and reduced markers of acute lung injury in LPS-treated rats [184].

High oxygen therapy (hyperoxia, >95% O_2_) is frequently used in the clinic as a therapy for acute, severe respiratory failure. In rodent models, hyperoxia (>95% O_2_) can cause ALI, associated with increased oxidative stress and lung inflammatory responses, leading to injury to respiratory endothelium and epithelium [185,186]. Hyperoxia induced ALI is characterized by neutrophil influx in the airways, pulmonary edema, pleural effusion, and increased lung cell apoptosis. In rats exposed to hyperoxia, HO-1 is markedly upregulated in lung epithelial, interstitial and inflammatory cells [43]. Gene transfer of *Hmox1* in rat lungs by intratracheal application of adenovirus, which increased HO-1 expression in the bronchiolar epithelium, protected against ALI and increased survival during hyperoxia exposure [187]. Consistently, adenoviral-directed HO-1 overexpression can protect A549 alveolar epithelial cells against hyperoxia-induced cell death [188].

Similar anti-inflammatory effects against hyperoxia-induced ALI, were observed in mice treated with iCO [189,190]. The administration of iCO (250 ppm) during hyperoxia exposure prolonged the survival of rats and mice subjected to a lethal exposure to hyperoxia, and reduced histological markers of lung injury, including airway neutrophil infiltration, fibrin deposition, alveolar proteinosis, pulmonary edema, and apoptosis, as compared to animals exposed to hyperoxia alone [189,190]. In mice, hyperoxia was shown to induce the expression of pro-inflammatory cytokines (i.e., TNF-α, IL-1β, IL-6) and activate major MAPK pathways in lung tissue. The protection afforded by CO treatment against the lethal effects of hyperoxia correlated with the inhibited release of pro-inflammatory cytokines in bronchioalveolar lavage (BAL). The protective effects of CO in the hyperoxia-induced ALI model required the MKK3/p38β MAPK pathway [190].

Mechanical ventilation can cause a type of ALI, termed ventilator-induced lung injury (VILI) in rodents associated with inflammation and airway neutrophil migration [191]. Rats ventilated with injurious (high tidal volume) ventilation in the presence of intraperitoneal LPS exhibited increased expression of HO-1 in the lung [192]. Mechanical ventilation at moderate tidal volume (12 mL/kg), in the absence of LPS, can also promote VILI in mice, and induce HO-1 protein expression in the lung [193]. CO application (e.g., 250 ppm) conferred protection in mouse VILI models by a mechanism primarily involving inhibition of neutrophil influx [193,194].

### 8.2. Sepsis

HO-1 has been implicated in host defense mechanisms during microbial sepsis, by mechanisms involving heme removal [30], and also by promoting bacterial clearance [195,196,197]. The HO-1 genetically deficient mice (*Hmox1*^−/−^) displayed a phenotype of sensitivity to polymicrobial sepsis, relative to wild-type mice. Transgenic mice overexpressing HO-1 in vascular smooth muscle cells and myofibroblasts protected against *Enterococcus faecalis* induced sepsis. The targeted increase in HO-1 expression did not inhibit influx of circulating inflammatory cells but augmented bacterial clearance by increasing phagocytosis and endogenous antimicrobial responses. Application of CO via CORM-2 improved mouse survival in this model [195].

Endogenous HO-1 may play a protective role in mitigating the systemic inflammatory response to sepsis [196]. Circulating levels of high mobility group box 1 (HMGB1) are increased as a late component of the systemic inflammatory response and have been implicated in sepsis-associated mortality. Lung inflammation, HMGB1 protein levels, and HMGB1 expression in inflammatory cells were increased in *Hmox1*^−/−^ mice relative to wild type mice. After LPS challenge, circulating levels of HMGB1 in *Hmox1*^−/−^ mice were higher relative to wild type mice; and these could be inhibited by pharmacological application of CO via CORM2 or BV. Furthermore, treatment with CO or BV, or with a HMGB1-neutralizing antibody improved survival of *Hmox1*^−/−^ mice subjected to LPS challenge. Thus, increased HMGB1 levels can aggravate LPS-induced mortality in *Hmox1*^−/−^ mice [196]. Heme treatment or application of CORM-2 significantly reduced plasma levels of HMGB1 in mice challenged with LPS or subjected to cecal-ligation and puncture (CLP)-induced polymicrobial sepsis, reduced serum TNF-α, and IL-1β levels, and increased survival [197]. Pretreatment with hemin or overexpression of HO-1 significantly inhibited HMGB1 release, translocation of HMGB1, and production of pro-inflammatory cytokines (i.e., TNF-α, IL-1β, and IFN-β) in RAW 264.7 cells stimulated with LPS. These effects were also achieved by administration of CO donor compounds and reversed by CO scavenging molecules (i.e., oxyhemoglobin). The authors concluded that HO-1-derived CO reduces HMGB1 release in LPS-activated cells [197].

Application of iCO (250 ppm) either prior to or after CLP improved mouse survival. The protective effects of CO in CLP were related to activation of Beclin-1-dependent autophagy and phagocytosis, reduced inflammation, and enhanced bacterial clearance from liver, lung, and blood [198]. In an *E. coli* infection model, endogenous HO-derived CO was associated with increased macrophage phagocytosis by a mechanism that involved inflammasome-dependent immune responses [199]. CO derived from CORM-3 was also demonstrated to confer cardioprotection in CLP-induced polymicrobial sepsis by downregulating NLRP3-dependent inflammasome activation [200]. 

### 8.3. Acute Kidney Injury (Sepsis and Ischemia/Reperfusion)

HO-1 expression has been associated with tissue protection in models of acute kidney injury (AKI) induced by sepsis and other insults such ischemia/reperfusion (I/R) and exposure to cisplatin [35,201]. Early studies recognized the potential of heme both as a mediator of kidney injury and as a means for therapeutic preconditioning to induce HO-1 [202]. *Hmox1*^−/−^ mice, which exhibit renal iron deposition, were sensitive to cisplatin-induced and glycerol-induced AKI [203,204]. Recent studies using myeloid-specific HO-1 deleted mice, demonstrated that these mice were susceptible to I/R-induced AKI with increased renal inflammation and apoptosis [205]. Protection from I/R derived by heme preconditioning was associated with increased HO-1 in CD11b^+^ F4/80^lo^ renal myeloid cells. Mitochondria-specific HO-1 targeting also protected cultured renal proximal epithelial cells against hypoxia-induced cytotoxicity and oxidative stress in vitro [206]. CO derived from CORM-2 can exert protection in sepsis-induced acute AKI in rats subjected to CLP [206], as evidenced by reduced serum creatinine and blood urea nitrogen levels, reduced kidney cell apoptosis, increased survival, and decreased renal injury scores. Treatment with CORM-2 reduced TNF-α and IL-1β levels and oxidative stress, and inhibited inflammasome-associated caspase-1 activation [207]. The therapeutic potential of CO and CO-releasing molecules for AKI has recently been summarized elsewhere [208]. 

### 8.4. Malaria

Malaria is a serious disease associated with *Plasmodium* infection. Malaria associated (MA)-ALI/ARDS is a major clinical complication of severe malaria, which is characterized by a high mortality rate and resistance to therapy. HO-1 expression occurs in inflammatory cells in severe human malaria and may represent a therapeutic target in animal models of experimental malaria [209]. DBA/2 mice infected with *Plasmodium berghei ANKA* (PbA) develop signs of ALI/ARDS that resemble the human disease, which include pulmonary edema, hemorrhage, pleural effusion, and hypoxemia. Increased pulmonary HO-1 expression was observed in mouse models of MA-ALI/ARDS [14,210]. These protective effects were attributed to HO-1 dependent degradation of free heme, a pro-inflammatory mediator in malaria [14,210]. Pharmacological heme treatment to induce HO-1 reduced MA-ALI/ARDS, improved respiratory function, reduced serum vascular endothelial growth factor (VEGF) levels, and improved vascular permeability in this model [210]. Furthermore, application of iCO (250 ppm, 72 h) prevented death of PbA-infected DBA/2 mice by ALI, associated with reduction of circulating VEGF. iCO administration (250 ppm) also reduced hemorrhage and pulmonary inflammation in this model [211]. 

In a mouse model of experimental cerebral malaria (ECM) resulting from infection with PbA, Balb/c mice express high levels of HO-1 in the brain after infection. *Hmox1*^−/−^ mice were sensitized to the lethal effects of ECM. HO-1 mediated protection in this model was associated with heme removal and anti-inflammatory effects due to the endogenous production of HO-derived CO [212]. Pharmacological upregulation of HO-1 in this model reduced blood-brain barrier disruption, brain microvasculature congestion and neuroinflammation including CD8(+) T-cell brain sequestration. Furthermore, iCO treatment (250 ppm) also conferred protection in PBA-induced ECM by similar mechanisms [29]. Pharmacological application of CO with experimental CORM (ALF492) also reduced neuroinflammation in ECM [212]. These studies, taken together, suggest that modulation iCO and/or pharmacological application of CORMs, may represent a potential therapeutic strategy for MA-ALI and ECM associated with *Plasmodium* infection.

## 9. Therapeutic Implications

Disorders of heme synthesis and degradation may lead to human diseases, which can include porphyrias, anemias, and hyperbilirubinemia. Treatments for acute porphyrias may include glucose or hematin infusions. Synthetic α-melanocyte stimulating hormone (α-MSH) (afamelanotide) is used for cutaneous porphyria to decrease skin photosensitivity [213]. Oral β-carotene is also used to decrease phototoxicity in cutaneous porphyria [214]. With respect to excess HO activity, phototherapy is commonly used for hyperbilirubinemia to oxidize excess BR.

Targeting the heme/heme oxygenase system may have therapeutic benefit in select human diseases in a context-specific fashion [1,40]. Unbound heme, a pro-oxidant molecule, has been implicated in the pathogenesis of diverse diseases, such as sepsis, malaria, SCD, and other hemolytic or inflammatory diseases, inclusive of cerebral hemorrhage and neurodegenerative disorders [1,13,30,119]. Nevertheless, pharmacological application of heme as a preconditioning agent to induce the HO-1 mediated stress response and other cellular responses, has been proposed experimentally for sepsis and I/R injury [1,40]. Therapeutic strategies targeting enhanced heme degradation may include the design or implementation of non-toxic inducers of HO-1 for therapeutic benefit, as well as compounds that may target the antioxidant response more broadly via activation of Nrf2 [215]. Application of HO-1 inhibitors has therapeutic potential under conditions where excess HO activity may present a threat, such as hyperbilirubinemia [73] or neurodegenerative disorders [141]. The therapeutic administration of HO-1 activity end-products continues to show promise as an experimental strategy to alleviate inflammation-mediated organ injuries. In this regard, application of gaseous CO and or bile pigments can show benefit in sepsis and organ I/R injury models. As an alternative to therapies based on gaseous CO, worldwide efforts continue to harness the therapeutic benefit of CO via the design, synthesis and testing of novel CO-releasing molecules (i.e., those with transition metal centers) or organic CO donor compounds [54,55]. Pilot clinical trials in iCO therapy to date have been largely focused on safety of human administration, for pulmonary indications such as sepsis-induced acute respiratory distress syndrome (ARDS), idiopathic pulmonary fibrosis (IPF), and chronic obstructive pulmonary disease [216,217,218]. To date, these efforts have been mainly directed toward safety of dose application, and await further expanded clinical trials to establish efficacy. Whether iCO or CORMs will provide safe and effective modalities for the treatment of human disease requires further research directed at understanding the pharmacokinetics and toxicology of CO application in humans, and further clinical efficacy trials for select indications.

## Data Availability

Not applicable.

## References

[1] Immenschuh S., Vijayan V., Janciauskiene S., Gueler F. (2017). Heme as a target for therapeutic interventions. Front. Pharmacol..

[2] Maines M.D. (1984). New developments in the regulation of heme metabolism and their implications. Crit. Rev. Toxicol..

[3] Abraham N.G., Lin J.H., Schwartzman M.L., Levere R.D., Shibahara S. (1988). The physiological significance of heme oxygenase. Int. J. Biochem..

[4] Maines M.D. (1997). The heme oxygenase system: A regulator of second messenger gases. Annu. Rev. Pharmacol. Toxicol..

[5] Taketani S. (2005). Aquisition, mobilization and utilization of cellular iron and heme: Endless findings and growing evidence of tight regulation. Tohoku J. Exp. Med..

[6] Donegan R.K., Moore C.M., Hanna D.A., Reddi A.R. (2019). Handling heme: The mechanisms underlying the movement of heme within and between cells. Free Radic. Biol. Med..

[7] Fujita H. (1997). Molecular mechanism of heme biosynthesis. Tohoku J. Exp. Med..

[8] Ponka P. (1999). Cell biology of heme. Am. J. Med. Sci..

[9] Tsiftsoglou A.S., Tsamadou A.I., Papadopoulou L.C. (2006). Heme as key regulator of major mammalian cellular functions: Molecular, cellular, and pharmacological aspects. Pharmacol. Ther..

[10] Wang B. (2021). The acute hepatic porphyrias. Transl. Gastroenterol. Hepatol..

[11] Dawe R. (2017). An overview of the cutaneous porphyrias. F1000Research.

[12] Ryter S.W., Tyrrell R.M. (2000). The heme synthesis and degradation pathways: Role in oxidant sensitivity. Heme oxygenase has both pro- and antioxidant properties. Free Radic. Biol. Med..

[13] Chiabrando D., Vinchi F., Fiorito V., Mercurio S., Tolosano E. (2014). Heme in pathophysiology: A matter of scavenging, metabolism and trafficking across cell membranes. Front. Pharmacol..

[14] Seixas E., Gozzelino R., Chora A., Ferreira A., Silva G., Larsen R., Rebelo S., Penido C., Smith N.R., Coutinho A. (2009). Heme oxygenase-1 affords protection against noncerebral forms of severe malaria. Proc. Natl. Acad. Sci. USA.

[15] Su X., Wang H., Lin Y., Chen F. (2018). RIP1 and RIP3 mediate hemin-induced cell death in HT22 hippocampal neuronal cells. Neuropsychiatr. Dis. Treat..

[16] Laird M.D., Wakade C., Alleyne C.H., Dhandapani K.M. (2008). Hemin-induced necroptosis involves glutathione depletion in mouse astrocytes. Free Radic. Biol. Med..

[17] Zille M., Karuppagounder S.S., Chen Y., Gough P.J., Bertin J., Finger J., Milner T.A., Jonas E.A., Ratan R.R. (2017). Neuronal death after hemorrhagic stroke in vitro and in vivo shares features of ferroptosis and necroptosis. Stroke.

[18] Fortes G.B., Alves L.S., de Oliveira R., Dutra F.F., Rodrigues D., Fernandez P.L., Souto-Padron T., De Rosa M.J., Kelliher M., Golenbock D. (2012). Heme induces programmed necrosis on macrophages through autocrine TNF and ROS production. Blood.

[19] Balla J., Jacob H.S., Balla G., Nath K., Eaton J.W., Vercellotti G.M. (1993). Endothelial-cell heme uptake from heme proteins: Induction of sensitization and desensitization to oxidant damage. Proc. Natl. Acad. Sci. USA.

[20] Balla J., Vercellotti G.M., Jeney V., Yachie A., Varga Z., Jacob H.S., Eaton J.W., Balla G. (2007). Heme, heme oxygenase, and ferritin: How the vascular endothelium survives (and dies) in an iron-rich environment. Antioxid. Redox Signal.

[21] Balla J., Vercellotti G.M., Jeney V., Yachie A., Varga Z., Eaton J.W., Balla G. (2005). Heme, heme oxygenase and ferritin in vascular endothelial cell injury. Mol. Nutr. Food Res..

[22] Jeney V., Balla J., Yachie A., Varga Z., Vercellotti G.M., Eaton J.W., Balla G. (2002). Pro-oxidant and cytotoxic effects of circulating heme. Blood.

[23] Telen M.J., Malik P., Vercellotti G.M. (2019). Therapeutic strategies for sickle cell disease: Towards a multi-agent approach. Nat. Rev. Drug Discov..

[24] Belcher J.D., Chen C., Nguyen J., Abdulla F., Zhang P., Nguyen H., Nguyen P., Killeen T., Miescher S.M., Brinkman N. (2018). Haptoglobin and hemopexin inhibit vaso-occlusion and inflammation in murine sickle cell disease: Role of heme oxygenase-1 induction. PLoS ONE.

[25] Belcher J.D., Chen C., Nguyen J., Milbauer L., Abdulla F., Alayash A.I., Smith A., Nath K.A., Hebbel R.P., Vercellotti G.M. (2014). Heme triggers TLR4 signaling leading to endothelial cell activation and vaso-occlusion in murine sickle cell disease. Blood.

[26] Belcher J.D., Beckman J.D., Balla G., Balla J., Vercellotti G. (2010). Heme degradation and vascular injury. Antioxid. Redox Signal.

[27] Gbotosho O.T., Kapetanaki M.G., Kato G.J. (2021). The worst things in life are free: The role of free heme in sickle cell disease. Front. Immunol..

[28] Ferreira A., Balla J., Jeney V., Balla G., Soares M.P. (2008). A central role for free heme in the pathogenesis of severe malaria: The missing link?. J. Mol. Med..

[29] Pamplona A., Ferreira A., Balla J., Jeney V., Balla G., Epiphanio S., Chora A., Rodrigues C.D., Gregoire I.P., Cunha-Rodrigues M. (2007). Heme oxygenase-1 and carbon monoxide suppress the pathogenesis of experimental cerebral malaria. Nat. Med..

[30] Larsen R., Gozzelino R., Jeney V., Tokaji L., Bozza F.A., Japiassú A.M., Bonaparte D., Cavalcante M.M., Chora A., Ferreira A. (2010). A central role for free heme in the pathogenesis of severe sepsis. Sci. Transl. Med..

[31] Ghosh S., Flage B., Weidert F., Ofori-Acquah S.F. (2019). P-selectin plays a role in haem-induced acute lung injury in sickle mice. Br. J. Haematol..

[32] Shaver C.M., Upchurch C.P., Janz D.R., Grove B.S., Putz N.D., Wickersham N.E., Dikalov S.I., Ware B., Bastarache J.A. (2016). Cell-free hemoglobin: A novel mediator of acute lung injury. Am. J. Physiol. Lung Cell Mol. Physiol..

[33] Nath K.A., Belcher J.D., Nath M.C., Grande J.P., Croatt A.J., Ackerman A.W., Katusic Z.S., Vercellotti G.M. (2018). Role of TLR4 signaling in the nephrotoxicity of heme and heme proteins. Am. J. Physiol. Renal Physiol..

[34] Tracz M.J., Alam J., Nath K.A. (2007). Physiology and pathophysiology of heme: Implications for kidney disease. J. Am. Soc. Nephrol..

[35] Nath M., Agarwal A. (2020). New insights into the role of heme oxygenase-1 in acute kidney injury. Kidney Res. Clin. Pract..

[36] Tenhunen R., Marver H.S., Schmid R. (1968). The enzymatic conversion of heme to bilirubin by microsomal heme oxygenase. Proc. Natl. Acad. Sci. USA.

[37] Tenhunen R., Marver H.S., Schmid R. (1969). Microsomal heme oxygenase. Characterization of the enzyme. J. Biol. Chem..

[38] Yoshida T., Migita C.T. (2000). Mechanism of heme degradation by heme oxygenase. J. Inorg. Biochem..

[39] Cruse I., Maines M.D. (1988). Evidence suggesting that the two forms of heme oxygenase are products of different genes. J. Biol Chem..

[40] Ryter S.W., Choi A.M. (2016). Targeting heme oxygenase-1 and carbon monoxide for therapeutic modulation of inflammation. Transl. Res..

[41] Keyse S.M., Tyrrell R.M. (1989). Heme oxygenase is the major 32-kDa stress protein induced in human skin fibroblasts by UVA radiation, hydrogen peroxide, and sodium arsenite. Proc. Natl. Acad. Sci. USA.

[42] Alam J., Shibahara S., Smith A. (1989). Transcriptional activation of the heme oxygenase gene by heme and cadmium in mouse hepatoma cells. J. Biol. Chem..

[43] Lee P.J., Alam J., Sylvester S.L., Inamdar N., Otterbein L., Choi A.M. (1996). Regulation of heme oxygenase-1 expression in vivo and in vitro in hyperoxic lung injury. Am. J. Respir. Cell. Mol. Biol..

[44] Lee P.J., Jiang B.H., Chin B.Y., Iyer N.V., Alam J., Semenza G.L., Choi A.M. (1997). Hypoxia-inducible factor-1 mediates transcriptional activation of the heme oxygenase-1 gene in response to hypoxia. J. Biol. Chem..

[45] Yachie A., Niida Y., Wada T., Igarashi N., Kaneda H., Toma T., Ohta K., Kasahara Y., Koizumi S. (1999). Oxidative stress causes enhanced endothelial cell injury in human heme oxygenase-1 deficiency. J. Clin. Investig..

[46] Poss K.D., Tonegawa S. (1997). Heme oxygenase-1 is required for mammalian iron reutilization. Proc. Natl. Acad. Sci. USA.

[47] Poss K.D., Tonegawa S. (1997). Reduced stress defense in heme oxygenase 1-deficient cells. Proc. Natl. Acad. Sci. USA.

[48] Otterbein L.E., Bach F.H., Alam J., Soares M., Tao Lu H., Wysk M., Davis R.J., Flavell R.A., Choi A.M. (2000). Carbon monoxide has anti-inflammatory effects involving the mitogen-activated protein kinase pathway. Nat. Med..

[49] Petrache I., Otterbein L.E., Alam J., Wiegand G.W., Choi A.M. (2000). Heme oxygenase-1 inhibits TNF-alpha-induced apoptosis in cultured fibroblasts. Am. J. Physiol. Lung Cell Mol. Physiol..

[50] Brouard S., Otterbein L.E., Anrather J., Tobiasch E., Bach F.H., Choi A.M., Soares M.P. (2000). Carbon monoxide generated by heme oxygenase-1 suppresses endothelial cell apoptosis. J. Exp. Med..

[51] Motterlini R., Otterbein L.E. (2010). The therapeutic potential of carbon monoxide. Nat. Rev. Drug Discov..

[52] Ryter S.W., Ma K.C., Choi A.M.K. (2018). Carbon monoxide in lung cell physiology and disease. Am. J. Physiol. Cell Physiol..

[53] Ryter S.W. (2020). Therapeutic potential of heme oxygenase-1 and carbon monoxide in acute organ injury, critical illness, and inflammatory disorders. Antioxidants.

[54] Ji X., Wang B. (2018). Strategies toward organic carbon monoxide prodrugs. Acc. Chem. Res..

[55] Ling K., Men F., Wang W.C., Zhou Y.Q., Zhang H.W., Ye D.W. (2018). Carbon monoxide and its controlled release: Therapeutic application, detection, and development of carbon monoxide releasing molecules (CORMs). J. Med. Chem..

[56] Siracusa R., Schaufler A., Calabrese V., Fuller P.M., Otterbein L.E. (2021). Carbon monoxide: From poison to clinical trials. Trends Pharmacol. Sci..

[57] Ogun A.S., Joy N.V., Valentine M. (2020). Biochemistry Heme Synthesis.

[58] Dailey H., Dailey T., Wu C.K., Medlock A.E., Wang K.F., Rose J.P., Wang B.C. (2000). Ferrochelatase at the millennium: Structures, mechanisms and [2Fe-2S] clusters. CMLS Cell. Mol. Life Sci..

[59] Swenson S.A., Moore C.M., Marcero J.R., Medlock A.E., Reddi A.R., Khalimonchuk O. (2020). From synthesis to utilization: The ins and outs of mitochondrial heme. Cells.

[60] Hederstedt L. (2012). Heme A biosynthesis. Biochim Biophys Acta (BBA)—Bioenergetics.

[61] Whatley S.D., Ducamp S., Gouya L., Grandchamp B., Beaumont C., Badminton M.N., Elder G.H., Holme S.A., Anstey A.V., Parker M. (2008). C-terminal deletions in the ALAS2 gene lead to gain of function and cause X-linked dominant protoporphyria without anemia or iron overload. Am. J. Hum. Genet..

[62] Fujiwara T., Harigae H. (2015). Biology of heme in mammalian erythroid cells and related disorders. Biomed. Res. Int..

[63] Tenhunen R., Ross M.E., Marver H.S., Schmid R. (1970). Reduced nicotinamide-adenine dinucleotide phosphate dependent biliverdin reductase: Partial purification and characterization. Biochemistry.

[64] Gazzin S., Vitek L., Watchko J., Shapiro S.M., Tiribelli C.A. (2016). Novel perspective on the biology of bilirubin in health and disease. Trends Mol. Med..

[65] Sticova E., Jirsa M. (2013). New insights in bilirubin metabolism and their clinical implications. World J. Gastroenterol..

[66] Takahashi S., Wang J., Rousseau D.L., Ishikawa K., Yoshida T., Host J.R., Ikeda-Saito M. (1994). Heme-heme oxygenase complex. Structure of the catalytic site and its implication for oxygen activation. J. Biol. Chem..

[67] McCoubrey W.K., Huang T.J., Maines M.D. (1997). Heme oxygenase-2 is a hemoprotein and binds heme through heme regulatory motifs that are not involved in heme catalysis. J. Biol. Chem..

[68] Liu L., Dumbrepatil A.B., Fleischhacker A.S., Marsh E.N.G., Ragsdale S.W. (2020). Heme oxygenase-2 is post-translationally regulated by heme occupancy in the catalytic site. J. Biol. Chem..

[69] Fleischhacker A.S., Gunawan A.L., Kochert B.A., Liu L., Wales T.E., Borowy M.C., Engen J.R., Ragsdale S.W. (2020). The heme-regulatory motifs of heme oxygenase-2 contribute to the transfer of heme to the catalytic site for degradation. J. Biol. Chem..

[70] Fleischhacker A.S., Carter E.L., Ragsdale S.W. (2018). Redox regulation of heme oxygenase-2 and the transcription factor, Rev-Erb, through heme regulatory motifs. Antioxid. Redox Signal.

[71] Muchowski K.E. (2014). Evaluation and treatment of neonatal hyperbilirubinemia. Am. Fam. Physician.

[72] Lin J.P., O’Donnell C.J., Schwaiger J.P., Cupples L.A., Lingenhel A., Hunt S.C., Yang S., Kronenberg F. (2006). Association between the UGT1A1*28 allele, bilirubin levels, and coronary heart disease in the Framingham Heart Study. Circulation.

[73] Itoh S., Okada H., Kuboi T., Kusaka T. (2017). Phototherapy for neonatal hyperbilirubinemia. Pediatr. Int..

[74] Wang J., Guo G., Li A., Cai W.Q., Wang X. (2021). Challenges of phototherapy for neonatal hyperbilirubinemia. Exp. Ther. Med..

[75] Suresh G.K., Martin C.L., Soll R.F. (2003). Metalloporphyrins for treatment of unconjugated hyperbilirubinemia in neonates. Cochrane Database Syst. Rev..

[76] Adamson J.W., Finch C.A. (1975). Hemoglobin function, oxygen affinity, and erythropoietin. Annu. Rev. Physiol..

[77] Evans S.V., Brayer G.D. (1988). Horse heart metmyoglobin. A 2.8-A resolution three-dimensional structure determination. J. Biol. Chem..

[78] Pesce A., Dewilde S., Nardini M., Moens L., Ascenzi P., Hankeln T., Burmester T., Bolognesi M. (2004). The human brain hexacoordinated neuroglobin three-dimensional structure. Micron.

[79] Kim H.J., Khalimonchuk O., Smith P.M., Winge D.R. (2012). Structure, function, and assembly of heme centers in mitochondrial respiratory complexes. Biochim. Biophys. Acta..

[80] DeWitt D.L., el-Harith E.A., Kraemer S.A., Andrews M.J., Yao E.F., Armstrong R.L., Smith W.L. (1990). The aspirin and heme-binding sites of ovine and murine prostaglandin endoperoxide synthases. J. Biol. Chem..

[81] Yan D., Lin Y.W., Tan X. (2017). Heme-containing enzymes and inhibitors for tryptophan metabolism. Metallomics.

[82] Förstermann U., Kleinert H. (1995). Nitric oxide synthase: Expression and expressional control of the three isoforms. Naunyn Schmiedebergs Arch. Pharmacol..

[83] Dioum E.M., Rutter J., Tuckerman J.R., Gonzalez G., Gilles-Gonzalez M.A., McKnight S.L. (2002). NPAS2: A gas-responsive transcription factor. Science.

[84] Raghuram S., Stayrook K.R., Huang P., Rogers P.M., Nosie A.K., McClure D.B., Burris L.L., Khorasanizadeh S., Burris T.P., Rastinejad F. (2007). Identification of heme as the ligand for the orphan nuclear receptors REV-ERBalpha and REV-ERBbeta. Nat. Struct. Mol. Biol..

[85] Wu N., Yin L., Hanniman E.A., Joshi S., Lazar M.A. (2009). Negative feedback maintenance of heme homeostasis by its receptor, Rev-erbalpha. Genes Dev..

[86] Igarashi K., Watanabe-Matsui M. (2014). Wearing red for signaling: The heme-bach axis in heme metabolism, oxidative stress response and iron immunology. Tohoku J. Exp. Med..

[87] Grigg J.C., Shumayrikh N., Sen D. (2014). G-quadruplex structures formed by expanded hexanucleotide repeat RNA and DNA from the neurodegenerative disease-linked C9orf72 gene efficiently sequester and activate heme. PLoS ONE.

[88] Shumayrikh N.M., Warren J.J., Bennet A.J., Sen D. (2021). A heme•DNAzyme activated by hydrogen peroxide catalytically oxidizes thioethers by direct oxygen atom transfer rather than by a Compound I-like intermediate. Nucleic Acids Res..

[89] Vinchi F., Ingoglia G., Chiabrando D., Mercurio S., Turco E., Silengo L., Altruda F., Tolosano E. (2014). Heme exporter FLVCR1a regulates heme synthesis and degradation and controls activity of cytochromes P450. Gastroenterology.

[90] Chiabrando D., Marro S., Mercurio S., Giorgi C., Petrillo S., Vinchi F., Fiorito V., Fagoonee S., Camporeale A., Turco E. (2012). The mitochondrial heme exporter FLVCR1b mediates erythroid differentiation. J. Clin. Investig..

[91] Tolosano E., Altruda F. (2002). Hemopexin: Structure, function, and regulation. DNA Cell Biol..

[92] Huang X., Groves J.T. (2018). Oxygen activation and radical transformations in heme proteins and metalloporphyrins. Chem. Rev..

[93] Koren R., Kremer M.L. (1969). Decomposition of H2O2 by haemin. Inhibition of the reaction by azide. Biochim. Biophys. Acta.

[94] George P. (1948). A comparison of the decomposition of hydrogen peroxide by catalase, ferrous and ferric ions, haemin and ferrous phthalocyanine. Biochem. J..

[95] Kumar S., Bandyopadhyay U. (2005). Free heme toxicity and its detoxification systems in human. Toxicol. Lett..

[96] Vincent S.H. (1989). Oxidative effects of heme and porphyrins on proteins and lipids. Semin. Hematol..

[97] Carlsen C.U., Møller J.K.S., Skibsted L.H. (2005). Heme-iron in lipid oxidation. Coordin. Chem Rev..

[98] Tappel A.L. (1955). Unsaturated lipide oxidation catalyzed by hematin compounds. J. Biol. Chem..

[99] Gutteridge J.M., Smith A. (1988). Antioxidant protection by haemopexin of haem-stimulated lipid peroxidation. Biochem. J..

[100] Thomas D.D., Espey M.G., Vitek M.P., Miranda K.M., Wink D.A. (2002). Protein nitration is mediated by heme and free metals through Fenton-type chemistry: An alternative to the NO/O2- reaction. Proc. Natl. Acad. Sci. USA.

[101] Grinshtein N., Bamm V.V., Tsemakhovich V.A., Shaklai N. (2003). Mechanism of low-density lipoprotein oxidation by hemoglobin-derived iron. Biochemistry.

[102] Balla G., Jacob H.S., Eaton J.W., Belcher J.D., Vercellotti G.M. (1991). Hemin: A possible physiological mediator of low-density lipoprotein oxidation and endothelial injury. Arterioscler. Thromb..

[103] Vercellotti G.M., Balla G., Balla J., Nath K., Eaton J.W., Jacob H.S. (1994). Heme and the vasculature: An oxidative hazard that induces antioxidant defenses in the endothelium. Artif. Cells Blood Substit. Immobil. Biotechnol..

[104] Dutra F.F., Bozza M.T. (2014). Heme on innate immunity and inflammation. Front. Pharmacol..

[105] Janciauskiene S., Vijayan V., Immenschuh S. (2020). TLR4 Signaling by heme and the role of heme-binding blood proteins. Front. Immunol..

[106] Wagener F.A., Eggert A., Boerman O.C., Oyen W.J., Verhofstad A., Abraham N.G., Adema G., van Kooyk Y., de Witte T., Figdor C.G. (2001). Heme is a potent inducer of inflammation in mice and is counteracted by heme oxygenase. Blood.

[107] Figueiredo R.T., Fernandez P.L., Mourao-Sa D.S., Porto B.N., Dutra F.F., Alves L.S., Oliveira M.F., Oliveira P.L., Graça-Souza A.V., Bozza M.T. (2007). Characterization of heme as activator of Toll-like receptor 4. J. Biol. Chem..

[108] Prestes E.B., Alves L.S., Rodrigues D.A.S., Dutra F.F., Fernandez P.L., Paiva C.N., Kagan J.C., Bozza M.T. (2020). Mitochondrial reactive oxygen species participate in signaling triggered by heme in macrophages and upon hemolysis. J. Immunol..

[109] Sudan K., Vijayan V., Madyaningrana K., Gueler F., Igarashi K., Foresti R., Motterlini R., Immenschuh S. (2019). TLR4 activation alters labile heme levels to regulate BACH1 and heme oxygenase-1 expression in macrophages. Free Radic. Biol. Med..

[110] Belcher J.D., Zhang P., Nguyen J., Kiser Z.M., Nath K.A., Hu J., Trent J.O., Vercellotti G.M. (2020). Identification of a heme activation site on the MD-2/TLR4 complex. Front. Immunol..

[111] Zhang P., Nguyen J., Abdulla F., Nelson A.T., Beckman J.D., Vercellotti G.M., Belcher J.D. (2021). Soluble MD-2 and heme in sickle cell disease plasma promote pro-inflammatory signaling in endothelial cells. Front. Immunol..

[112] Dutra F.F., Alves L.S., Rodrigues D., Fernandez P.L., de Oliveira R.B., Golenbock D.T., Zamboni D.S., Bozza M.T. (2014). Hemolysis-induced lethality involves inflammasome activation by heme. Proc. Natl. Acad. Sci. USA.

[113] Bolívar B.E., Brown-Suedel A.N., Rohrman B.A., Charendoff C.I., Yazdani V., Belcher J.D., Vercellotti G.M., Flanagan J.M., Bouchier-Hayes L. (2021). Noncanonical roles of Caspase-4 and Caspase-5 in heme-driven IL-1β release and cell death. J. Immunol..

[114] Erdei J., Tóth A., Balogh E., Nyakundi B.B., Bányai E., Ryffel B., Paragh G., Cordero M.D., Jeney V. (2018). Induction of NLRP3 inflammasome activation by heme in human endothelial cells. Oxid. Med. Cell Longev..

[115] May O., Yatime L., Merle N.S., Delguste F., Howsam M., Daugan M.V., Paul-Constant C., Billamboz M., Ghinet A., Lancel S. (2020). The receptor for advanced glycation end products is a sensor for cell-free heme. FEBS J..

[116] James J., Srivastava A., Varghese. M.V., Eccles C.A., Zemskova M., Rafikova O., Rafikov R. (2020). Heme induces rapid endothelial barrier dysfunction via the MKK3/p38MAPK axis. Blood.

[117] Santaterra V., Fiusa M., Hounkpe B.W., Chenou F., Tonasse W.V., da Costa L., Garcia-Weber D., Domingos I.F., de Lima F., Borba-Junior I.T. (2020). Endothelial barrier integrity is disrupted *in vitro* by heme and by serum from sickle cell Disease Patients. Front. Immunol..

[118] Oishi S., Tsukiji N., Otake S., Oishi N., Sasaki T., Shirai T., Yoshikawa Y., Takano K., Shinmori H., Inukai T. (2021). Heme activates platelets and exacerbates rhabdomyolysis-induced acute kidney injury via CLEC-2 and GPVI/FcRγ. Blood Adv..

[119] Lin S., Yin Q., Zhong Q., Lv F.L., Zhou Y., Li J.Q., Wang J.Z., Su B.Y., Yang Q.W. (2012). Heme activates TLR4-mediated inflammatory injury via MyD88/TRIF signaling pathway in intracerebral hemorrhage. J. Neuroinflammation.

[120] Beckman J.D., Abdullah F., Chen C., Kirchner R., Rivera-Rodriguez D., Kiser Z.M., Nguyen A., Zhang P., Nguyen J., Hebbel R.P. (2021). Endothelial TLR4 expression mediates vaso-occlusive crisis in sickle cell disease. Front. Immunol..

[121] Chiabrando D., Fiorito V., Petrillo S., Tolosano E. (2018). Unraveling the role of heme in neurodegeneration. Front. Neurosci..

[122] Majno G., Joris I. (1995). Apoptosis, oncosis, and necrosis. An overview of cell death. Am. J. Pathol..

[123] Ryter S.W., Kim H.P., Hoetzel A., Park J.W., Nakahira K., Wang X., Choi A.M. (2007). Mechanisms of cell death in oxidative stress. Antioxid. Redox Signal..

[124] Linkermann A., Green D.R. (2014). Necroptosis. N. Engl. J. Med..

[125] Choi M.E., Price D.R., Ryter S.W., Choi A.M.K. (2019). Necroptosis: A crucial pathogenic mediator of human disease. JCI Insight..

[126] Dixon S.J., Lemberg K.M., Lamprecht M.R., Skouta R., Zaitsev E.M., Gleason C.E., Patel D.N., Bauer A.J., Cantley A.M., Yang W.S. (2012). Ferroptosis: An iron-dependent form of nonapoptotic cell death. Cell.

[127] Kwon M.Y., Park E., Lee S.J., Chung S.W. (2015). Heme oxygenase-1 accelerates erastin-induced ferroptotic cell death. Oncotarget.

[128] Chang L.C., Chiang S.K., Chen S.E., Yu Y.L., Chou R.H., Chang W.C. (2018). Heme oxygenase-1 mediates BAY 11–7085 induced ferroptosis. Cancer Lett..

[129] Guerrero-Hue M., García-Caballero C., Palomino-Antolín A., Rubio-Navarro A., Vázquez-Carballo C., Herencia C., Martín-Sanchez D., Farré-Alins V., Egea J., Cannata P. (2019). Curcumin reduces renal damage associated with rhabdomyolysis by decreasing ferroptosis-mediated cell death. FASEB J..

[130] Adedoyin O., Boddu R., Traylor A., Lever J.M., Bolisetty S., George J.F., Agarwal A. (2018). Heme oxygenase-1 mitigates ferroptosis in renal proximal tubule cells. Am. J. Physiol. Renal Physiol..

[131] Otterbein L.E., Choi A.M. (2000). Heme oxygenase: Colors of defense against cellular stress. Am. J. Physiol. Lung Cell. Mol. Physiol..

[132] Halliwell B., Gutteridge J.M. (1984). Oxygen toxicity, oxygen radicals, transition metals and disease. Biochem. J..

[133] Dröge W. (2002). Free radicals in the physiological control of cell function. Physiol. Rev..

[134] Vile G.F., Tyrrell R.M. (1993). Oxidative stress resulting from ultraviolet A irradiation of human skin fibroblasts leads to a heme oxygenase-dependent increase in ferritin. J. Biol. Chem..

[135] Vile G.F., Basu-Modak S., Waltner C., Tyrrell R.M. (1994). Heme oxygenase 1 mediates an adaptive response to oxidative stress in human skin fibroblasts. Proc. Natl. Acad. Sci. USA.

[136] Arosio P., Levi S. (2002). Ferritin, iron homeostasis, and oxidative damage. Free Radic. Biol. Med..

[137] Balla G., Jacob H.S., Balla J., Rosenberg M., Nath K., Apple F., Eaton J.W., Vercellotti G.M. (1992). Ferritin: A cytoprotective antioxidant strategem of endothelium. J. Biol. Chem..

[138] Juckett M.B., Balla J., Balla G., Jessurun J., Jacob H.S., Vercellotti G.M. (1995). Ferritin protects endothelial cells from oxidized low-density lipoprotein in vitro. Am. J. Pathol..

[139] Balla J., Nath K.A., Balla G., Juckett M.B., Jacob H.S., Vercellotti G.M. (1995). Endothelial cell heme oxygenase and ferritin induction in rat lung by hemoglobin in vivo. Am. J. Physiol..

[140] Ferris C.D., Jaffrey S.R., Sawa A., Takahashi M., Brady S.D., Barrow R.K., Tysoe S.A., Wolosker H., Barañano D.E., Doré S. (1999). Haem oxygenase-1 prevents cell death by regulating cellular iron. Nat. Cell Biol..

[141] Schipper H.M., Song W., Tavitian A., Cressatti M. (2019). The sinister face of heme oxygenase-1 in brain aging and disease. Prog. Neurobiol..

[142] Song W., Zukor H., Lin S.H., Liberman A., Tavitian A., Mui J., Vali H., Fillebeen C., Pantopoulos K., Wu T.D. (2012). Unregulated brain iron deposition in transgenic mice over-expressing HMOX1 in the astrocytic compartment. J. Neurochem..

[143] Gupta A., Lacoste B., Pistell P.J., Ingram D.K., Hamel E., Alaoui-Jamali M.A., Szarek W.A., Vlahakis J.Z., Jie S., Song W. (2014). Neurotherapeutic effects of novel HO-1 inhibitors in vitro and in a transgenic mouse model of Alzheimer’s disease. J. Neurochem..

[144] Vanella L., Barbagallo I., Tibullo D., Forte S., Zappalà A., Li Volti G. (2016). The non-canonical functions of the heme oxygenases. Oncotarget.

[145] Dennery P.A. (2014). Signaling function of heme oxygenase proteins. Antioxid. Redox Signal.

[146] Stocker R., Yamamoto Y., McDonagh A.F., Glazer A.N., Ames B.N. (1987). Bilirubin is an antioxidant of possible physiological importance. Science.

[147] Neuzil J., Stocker R. (1993). Bilirubin attenuates radical-mediated damage to serum albumin. FEBS Lett..

[148] Stocker R., Glazer A.N., Ames B.N. (1987). Antioxidant activity of albumin-bound bilirubin. Proc. Natl. Acad. Sci. USA.

[149] Stocker R., Ames B.N. (1987). Potential role of conjugated bilirubin and copper in the metabolism of lipid peroxides in bile. Proc. Natl. Acad. Sci. USA.

[150] Lee J., Kim E.J., Lee J.G., Kim B.S., Huh K.H., Kim M.S., Kim S.I., Kim Y.S., Joo D.J. (2021). Clinical impact of serum bilirubin levels on kidney transplant outcomes. Sci. Rep..

[151] Suh S., Cho Y.R., Park M.K., Kim D.K., Cho N.H., Lee M.K. (2018). Relationship between serum bilirubin levels and cardiovascular disease. PLoS ONE.

[152] Kapitulnik J., Maines M.D. (2009). Pleiotropic functions of biliverdin reductase: Cellular signaling and generation of cytoprotective and cytotoxic bilirubin. Trends Pharmacol. Sci..

[153] Florczyk U.M., Jozkowicz A., Dulak J. (2008). Biliverdin reductase: New features of an old enzyme and its potential therapeutic significance. Pharmacol. Rep..

[154] Wegiel B., Otterbein L.E. (2012). Go green: The anti-inflammatory effects of biliverdin reductase. Front. Pharmacol..

[155] Alam J., Stewart D., Touchard C., Boinapally S., Choi A.M., Cook J.L. (1999). Nrf2, a Cap’n’Collar transcription factor, regulates induction of the heme oxygenase-1 gene. J. Biol. Chem..

[156] Tonelli C., Chio I.I.C., Tuveson D.A. (2018). Transcriptional regulation by Nrf2. Antioxid. Redox Signal.

[157] Kang M.I., Kobayashi A., Wakabayashi N., Kim S.G., Yamamoto M. (2004). Scaffolding of Keap1 to the actin cytoskeleton controls the function of Nrf2 as key regulator of cytoprotective phase 2 genes. Proc. Natl. Acad. Sci. USA.

[158] Zipper L.M., Mulcahy R.T. (2002). The Keap1 BTB/POZ dimerization function is required to sequester Nrf2 in cytoplasm. J. Biol. Chem..

[159] Itoh K., Wakabayashi N., Katoh Y., Ishii T., O’Connor T., Yamamoto M. (2003). Keap1 regulates both cytoplasmic-nuclear shuttling and degradation of Nrf2 in response to electrophiles. Genes Cells.

[160] Itoh K., Wakabayashi N., Katoh Y., Ishii T., Igarashi K., Engel J.D., Yamamoto M. (1999). Keap1 represses nuclear activation of antioxidant responsive elements by Nrf2 through binding to the amino-terminal Neh2 domain. Genes Dev..

[161] Alam J., Killeen E., Gong P., Naquin R., Hu B., Stewart D., Ingelfinger J.R., Nath K.A. (2003). Heme activates the heme oxygenase-1 gene in renal epithelial cells by stabilizing Nrf2. Am. J. Physiol. Renal Physiol..

[162] Igarashi K., Sun J. (2006). The heme-Bach1 pathway in the regulation of oxidative stress response and erythroid differentiation. Antioxid. Redox Signal.

[163] Sun J., Hoshino H., Takaku K., Nakajima O., Muto A., Suzuki H., Tashiro S., Takahashi S., Shibahara S., Alam J. (2002). Hemoprotein Bach1 regulates enhancer availability of heme oxygenase-1 gene. EMBO J..

[164] Ogawa K., Sun J., Taketani S., Nakajima O., Nishitani C., Sassa S., Hayashi N., Yamamoto M., Shibahara S., Fujita H. (2001). Heme mediates derepression of Maf Recognition element through direct binding to transcription repressor Bach1. EMBO J..

[165] Oyake T., Itoh K., Motohashi H., Hayashi N., Hoshino H., Nishizawa M., Yamamoto M., Igarashi K. (1996). Bach proteins belong to a novel family of BTB-basic leucine zipper transcription factors that interact with MafK and regulate transcription through the NF-E2 site. Mol. Cell Biol..

[166] Sun J., Brand M., Zenke Y., Tashiro S., Groudine M., Igarashi K. (2004). Heme regulates the dynamic exchange of Bach1 and NF-E2-related factors in the Maf transcription factor network. Proc. Natl. Acad. Sci. USA.

[167] Alam J., Cai J., Smith A. (1994). Isolation and characterization of the mouse heme oxygenase-1 gene. Distal 5’ sequences are required for induction by heme or heavy metals. J. Biol. Chem..

[168] Alam J., Camhi S., Choi A.M. (1995). Identification of a second region upstream of the mouse heme oxygenase-1 gene that functions as a basal level and inducer-dependent transcription enhancer. J. Biol. Chem..

[169] Paine A., Eiz-Vesper B., Blasczyk R., Immenschuh S. (2010). Signaling to heme oxygenase-1 and its anti-inflammatory therapeutic potential. Biochem. Pharmacol..

[170] Alam J., Cook J.L. (2007). How many transcription factors does it take to turn on the heme oxygenase-1 gene?. Am. J. Respir. Cell Mol. Biol..

[171] Alam J., Igarashi K., Immenschuh S., Shibahara S., Tyrrell R.M. (2004). Regulation of heme oxygenase-1 gene transcription: Recent advances and highlights from the International Conference (Uppsala, 2003) on Heme Oxygenase. Antioxid. Redox Signal.

[172] Cheng X., Ku C.H., Siow R.C. (2013). Regulation of the Nrf2 antioxidant pathway by microRNAs: New players in micromanaging redox homeostasis. Free Radic. Biol. Med..

[173] Ryter S.W. (2021). Heme oxgenase-1, a cardinal modulator of regulated cell death and inflammation. Cells.

[174] Campbell N.K., Fitzgerald H.K., Dunne A. (2021). Regulation of inflammation by the antioxidant haem oxygenase 1. Nat. Rev. Immunol..

[175] Willis D., Moore A.R., Frederick R., Willoughby D.A. (1996). Heme oxygenase: A novel target for the modulation of the inflammatory response. Nat. Med..

[176] Matute-Bello G., Frevert C.W., Martin T.R. (2008). Animal models of acute lung injury. Am. J. Physiol. Lung Cell Mol. Physiol..

[177] Kitamura Y., Hashimoto S., Mizuta N., Kobayashi A., Kooguchi K., Fujiwara I., Nakajima H. (2001). Fas/FasL-dependent apoptosis of alveolar cells after lipopolysaccharide-induced lung injury in mice. Am. J. Respir. Crit. Care Med..

[178] Camhi S.L., Alam J., Otterbein L., Sylvester S.L., Choi A.M. (1995). Induction of heme oxygenase-1 gene expression by lipopolysaccharide is mediated by AP-1 activation. Am. J. Respir. Cell Mol. Biol..

[179] Otterbein L., Sylvester S.L., Choi A.M. (1995). Hemoglobin provides protection against lethal endotoxemia in rats: The role of heme oxygenase-1. Am. J. Respir. Cell Mol. Biol..

[180] Tamion F., Richard V., Renet S., Thuillez C. (2006). Protective effects of heme-oxygenase expression against endotoxic shock: Inhibition of tumor necrosis factor-alpha and augmentation of interleukin-10. J. Trauma.

[181] Inoue S., Suzuki M., Nagashima Y., Suzuki S., Hashiba T., Tsuburai T., Ikehara K., Matsuse T., Ishigatsubo Y. (2001). Transfer of heme oxygenase 1 cDNA by a replication-deficient adenovirus enhances interleukin 10 production from alveolar macrophages that attenuates lipopolysaccharide-induced acute lung injury in mice. Hum. Gene Ther..

[182] Fredenburgh L.E., Baron R.M., Carvajal I.M., Mouded M., Macias A.A., Ith B., Perrella M.A. (2005). Absence of heme oxygenase-1 expression in the lung parenchyma exacerbates endotoxin-induced acute lung injury and decreases surfactant protein-B levels. Cell Mol. Biol..

[183] Sarady J.K., Zuckerbraun B.S., Bilban M., Wagner O., Usheva A., Liu F., Ifedigbo E., Zamora R., Choi A.M., Otterbein L.E. (2004). Carbon monoxide protection against endotoxic shock involves reciprocal effects on iNOS in the lung and liver. FASEB J..

[184] Sarady-Andrews J.K., Liu F., Gallo D., Nakao A., Overhaus M., Ollinger R., Choi A.M., Otterbein L.E. (2005). Biliverdin administration protects against endotoxin-induced acute lung injury in rats. Am. J. Physiol. Lung Cell Mol. Physiol..

[185] Lee P.J., Choi A.M.K. (2003). Pathways of cell signaling in hyperoxia. Free Rad. Biol. Med..

[186] Crapo J.D. (1986). Morphologic changes in pulmonary oxygen toxicity. Annu. Rev. Physiol..

[187] Otterbein L.E., Kolls J.K., Mantell L.L., Cook J.L., Alam J., Choi A.M. (1999). Exogenous administration of heme oxygenase-1 by gene transfer provides protection against hyperoxic lung injury. J. Clin. Investig..

[188] Lee P.J., Alam J., Wiegand G.W., Choi A.M. (1996). Overexpression of heme oxygenase-1 in human pulmonary epithelial cells results in cell growth arrest and increased resistance to hyperoxia. Proc. Natl. Acad. Sci. USA.

[189] Otterbein L.E., Mantell L.L., Choi A.M. (1999). Carbon monoxide provides protection against hyperoxic lung injury. Am. J. Physiol..

[190] Otterbein L.E., Otterbein S.L., Ifedigbo E., Liu F., Morse D.E., Fearns C., Ulevitch R.J., Knickelbein R., Flavell R.A., Choi A.M. (2003). MKK3 mitogen-activated protein kinase pathway mediates carbon monoxide-induced protection against oxidant-induced lung injury. Am. J. Pathol..

[191] Slutsky A.S. (1999). Lung injury caused by mechanical ventilation. Chest.

[192] Dolinay T., Szilasi M., Liu M., Choi A.M. (2004). Inhaled carbon monoxide confers antiinflammatory effects against ventilator-induced lung injury. Am. J. Respir. Crit Care Med..

[193] Hoetzel A., Dolinay T., Vallbracht S., Zhang Y., Kim H.P., Ifedigbo E., Alber S., Kaynar A.M., Schmidt R., Ryter S.W. (2008). Carbon monoxide protects against ventilator-induced lung injury via PPAR-gamma and inhibition of Egr-1. Am. J. Respir. Crit. Care Med..

[194] Hoetzel A., Schmidt R., Vallbracht S., Goebel U., Dolinay T., Kim H.P., Ifedigbo E., Ryter S.W., Choi A.M. (2009). Carbon monoxide prevents ventilator-induced lung injury via caveolin-1. Crit. Care Med..

[195] Chung S.W., Liu X., Macias A.A., Baron R.M., Perrella M.A. (2008). Heme oxygenase-1-derived carbon monoxide enhances the host defense response to microbial sepsis in mice. J. Clin. Investig..

[196] Takamiya R., Hung C.C., Hall S.R., Fukunaga K., Nagaishi T., Maeno T., Owen C., Macias A.A., Fredenburgh L.E., Ishizaka A. (2009). High-mobility group box 1 contributes to lethality of endotoxemia in heme oxygenase-1-deficient mice. Am. J. Respir. Cell Mol. Biol..

[197] Tsoyi K., Lee T.Y., Lee Y.S., Kim H.J., Seo H.G., Lee J.H., Chang K.C. (2009). Heme-oxygenase-1 induction and carbon monoxide-releasing molecule inhibit lipopolysaccharide (LPS)-induced high-mobility group box 1 release in vitro and improve survival of mice in LPS- and cecal ligation and puncture-induced sepsis model in vivo. Mol. Pharmacol..

[198] Lee S., Lee S.J., Coronata A.A., Fredenburgh L.E., Chung S.W., Perrella M.A., Nakahira K., Ryter S.W., Choi A.M. (2014). Carbon monoxide confers protection in sepsis by enhancing beclin 1-dependent autophagy and phagocytosis. Antioxid. Redox Signal.

[199] Wegiel B., Larsen R., Gallo D., Chin B.Y., Harris C., Mannam P., Kaczmarek E., Lee P.J., Zuckerbraun B.S., Flavell R. (2014). Macrophages sense and kill bacteria through carbon monoxide-dependent inflammasome activation. J. Clin. Investig..

[200] Zhang W., Tao A., Lan T., Cepinskas G., Kao R., Martin C.M., Rui T. (2017). Carbon monoxide releasing molecule-3 improves myocardial function in mice with sepsis by inhibiting NLRP3 inflammasome activation in cardiac fibroblasts. Basic Res. Cardiol..

[201] Bolisetty S., Zarjou A., Agarwal A. (2017). Heme oxygenase 1 as a therapeutic target in acute kidney injury. Am. J. Kidney Dis..

[202] Maines M.D., Mayer R.D., Ewing J.F., McCoubrey W.K. (1993). Induction of kidney heme oxygenase-1 (HSP32) mRNA and protein by ischemia/reperfusion: Possible role of heme as both promotor of tissue damage and regulator of HSP32. J. Pharmacol. Exp. Ther..

[203] Shiraishi F., Curtis L.M., Truong L., Poss K., Visner G.A., Madsen K.M., Nick H.S., Agarwal A. (2000). Heme oxygenase-1 gene ablation or overexpression modulates cisplatin-induced renal tubular apoptosis and necrosis. Am. J. Physiol..

[204] Nath K.A., Haggard J.J., Croatt A.J., Grande J.P., Poss K.D., Alam J. (2000). The indispensability of heme oxygenase-1 in protecting against acute heme protein-induced toxicity in vivo. Am. J. Pathol..

[205] Rossi M., Thierry A., Delbauve S., Preyat N., Soares M.P., Roumeguère T., Leo O., Flamand V., Le Moine A., Hougardy J.M. (2017). Specific expression of heme oxygenase-1 by myeloid cells modulates renal ischemia-reperfusion injury. Sci. Rep..

[206] Bolisetty S., Traylor A., Zarjou A., Johnson M.S., Benavides G.A., Ricart K., Boddu R., Moore R.D., Landar A., Barnes S. (2013). Mitochondria-targeted heme oxygenase-1 decreases oxidative stress in renal epithelial cells. Am. J. Physiol. Renal. Physiol..

[207] Wang P., Huang J., Li Y., Ruiming C., Haidong W., Jiali L., Zitong H. (2015). Exogenous carbon monoxide decreases sepsis-induced acute kidney injury and inhibits NLRP3 inflammasome activation in rats. Int. J. Mol. Sci..

[208] Yang X., de Caestecker M., Otterbein L.E., Wang B. (2020). Carbon monoxide: An emerging therapy for acute kidney injury. Med. Res. Rev..

[209] Pereira M.L.M., Marinho C.R.F., Epiphanio S. (2018). Could heme oxygenase-1 be a new target for therapeutic intervention in malaria-associated acute lung injury/acute respiratory distress syndrome?. Front. Cell Infect. Microbiol..

[210] Pereira M.L., Ortolan L.S., Sercundes M.K., Debone D., Murillo O., Lima F.A., Marinho C.R., Epiphanio S. (2016). Association of heme oxygenase 1 with lung protection in malaria-associated ALI/ARDS. Mediators Inflamm..

[211] Epiphanio S., Campos M.G., Pamplona A., Carapau D., Pena A.C., Ataíde R., Monteiro C.A., Félix N., Costa-Silva A., Marinho C.R. (2010). VEGF promotes malaria-associated acute lung injury in mice. PLoS Pathog..

[212] Pena A.C., Penacho N., Mancio-Silva L., Neres R., Seixas J.D., Fernandes A.C., Romão C.C., Mota M.M., Bernardes G.J., Pamplona A. (2012). A novel carbon monoxide-releasing molecule fully protects mice from severe malaria. Antimicrob. Agents Chemother..

[213] Langendonk J.G., Manisha B., Karl E.A., Herbert L.B., Alexander V.A., Montgomery B.D., Joseph B., Chris E., Norbert J.N., Charles P. (2015). Afamelanotide for erythropoietic protoporphyria. N. Engl. J. Med..

[214] Thapar M., Bonkovsky H.L. (2008). The diagnosis and management of erythropoietic protoporphyria. Gastroenterol. Hepatol..

[215] Drummond G.S., Baum J., Greenberg M., Lewis D., Abraham N.G. (2019). HO-1 overexpression and underexpression: Clinical implications. Arch. Biochem Biophys..

[216] Fredenburgh L.E., Perrella M.A., Barragan-Bradford D., Hess D.R., Peters E., Welty-Wolf K.E., Kraft B.D., Harris R.S., Maurer R., Nakahira K. (2018). A phase I trial of low-dose inhaled carbon monoxide in sepsis-induced ARDS. JCI Insight.

[217] Rosas I.O., Rosas I.O., Goldberg H.J., Collard H.R., El-Chemaly S., Flaherty K., Hunninghake G.M., Lasky J.A., Lederer D.J., Machado R. (2018). A Phase II clinical trial of low-dose inhaled carbon monoxide in idiopathic pulmonary fibrosis. Chest.

[218] Bathoorn E., Slebos D.J., Postma D.S., Koeter G.H., van Oosterhout A.J., van der Toorn M., Boezen H.M., Kerstjens H.A. (2007). Anti-inflammatory effects of inhaled carbon monoxide in patients with COPD: A pilot study. Eur. Respir. J..

